# A Small RNA-Mediated Regulatory Network in *Arabidopsis thaliana* Demonstrates Connectivity Between phasiRNA Regulatory Modules and Extensive Co-Regulation of Transcription by miRNAs and phasiRNAs

**DOI:** 10.3389/fpls.2019.01710

**Published:** 2020-01-29

**Authors:** Jose A. Vargas-Asencio, Keith L. Perry

**Affiliations:** Plant Pathology and Plant-Microbe Biology Section, School of Integrative Plant Science, Cornell University, Ithaca, NY, United States

**Keywords:** Arabidopsis, miRNA, phasiRNA, network, regulation, degradome

## Abstract

Gene regulation involves the orchestrated action of multiple regulators to fine-tune the expression of genes. Hierarchical interactions and co-regulation among regulators are commonly observed in biological systems, leading to complex regulatory networks. Small RNA (sRNAs) have been shown to be important regulators of gene expression due to their involvement in multiple cellular processes. In plants, microRNA (miRNAs) and phased small interfering RNAs (phasiRNAs) correspond to two well-characterized types of sRNAs involved in the regulation of posttranscriptional gene expression, although information about their targets and interactions with other gene expression regulators is limited. We describe an extended sRNA-mediated regulatory network in *Arabidopsis thaliana* that provides a reference frame to understand sRNA biogenesis and activity at the genome-wide level. This regulatory network combines a comprehensive evaluation of phasiRNA production and sRNA targets supported by degradome data. The network includes ~17% of genes in the *A. thaliana* genome, representing ~50% annotated gene ontology (GO) functional categories. Approximately 14% of genes with GO annotations corresponding to regulation of gene expression were found to be under sRNA control. The unbiased bioinformatic approach used to produce the network was able to detect 107 *PHAS* loci (regions of phasiRNA production), 5,047 active phasiRNAs (~70% of which were non-canonical), and reconstruct 17 regulatory modules resulting from complex regulatory interactions between different sRNA-regulatory pathways. Known regulatory modules like miR173-TAS-PPR/TPR and miR390-TAS3-ARF/F-box were faithfully reconstructed and expanded, illustrating the accuracy and sensitivity of the methods and providing confidence for the validity of findings of previously unrecognized modules. The network presented here includes a 2X increase in the number of identified *PHAS* loci, a large complement (~70%) of non-canonical phasiRNAs, and the most comprehensive evaluation of sRNA cleavage activity in *A. thaliana* to date. Structural analysis showed similarities to networks of other biological systems and demonstrated connectivity between phasiRNA regulatory modules with extensive co-regulation of transcripts by miRNAs and phasiRNAs. The described regulatory network provides a reference that will facilitate global analyses of individual plant regulatory programs such as those that control homeostasis, development, and responses to biotic and abiotic environmental changes.

## Introduction

Gene expression regulation is a cellular process that involves the orchestrated action of multiple regulators to fine-tune the expression of genes (Walczak and Tkačik, 2011). It can be thought of as the sum of interactions between regulatory factors and their substrates across multiple levels, as well as the effect of cross regulation between regulators from different levels. Regulatory levels range from DNA availability (chromatin structure, methylation status) to RNA abundance and translational efficiency (Walczak and Tkačik, 2011). This complex, multilevel process can be more adequately represented and studied using network theories (Cumbo et al., 2014). Network-based approaches allow the investigation of biological features that emerge when regulatory systems are studied from a multiscale, genomic approach (Stumpf and Wiuf, 2009). Features such as the topology and dynamics of these networks have been proposed to be informative and provide insights into the way organisms function, develop, and respond to internal and external stimuli (Cora et al., 2017). Network concepts and applications of network principles to biological systems have been thoroughly reviewed (Albert, 2005; Zhu et al., 2007; Pavlopoulos et al., 2011).

Transcription factors and small RNAs (sRNAs) are considered to be the primary levels of gene expression regulation (Cora et al., 2017). They act in combination to form genetic regulatory circuits involved in transcriptional control (Megraw et al., 2016; Cora et al., 2017). A significant body of research is dedicated to understanding how transcription factors are involved in the regulation of multiple cellular processes in *Arabidopsis thaliana*, one of the best studied plant model systems (Taylor-Teeples et al., 2015; González-Morales et al., 2016; Drapek et al., 2017). In contrast, broad scale studies of sRNA-mediated regulation in *A. thaliana* are not common and their scope is limited due to challenges posed by the high false positive rate of bioinformatic predictions of small RNA activities (Ding et al., 2012).

sRNAs have been shown to be involved in higher level regulatory interactions by controlling the expression of other regulators (transcription factors, sRNA biogenesis factors), thereby extending their regulatory contribution by affecting downstream events (Wang and Chekanova, 2016; Cora et al., 2017). Because of this and their involvement in key cellular processes, several studies propose them to be "master regulators" of gene expression and phenotype determination (Sun et al., 2010; Voorhoeve, 2010; Zhai et al., 2011).

In plants, sRNAs are the products of multiple biogenesis pathways (Borges and Martienssen, 2015). Because of their broad regulatory potential and better understood biogenesis and mode of actions (Wang and Chekanova, 2016), most reports focus on microRNAs (miRNAs) and phased interfering small RNAs (phasiRNAs). miRNAs are the better studied example, as their biogenesis and activity have been investigated intensively, and their involvement in multiple cellular processes through post transcriptional gene silencing (PTGS) has been well established (Borges and Martienssen, 2015). phasiRNAs are sRNAs produced from multiple pathways whose regulation involves regulatory cascades or modules; phasiRNAs are usually derived from a miRNA-transcript targeting event, leading to the production of additional phased small RNAs with the potential to regulate gene expression in cis and trans. sRNAs that induce phasiRNA production are referred to as "triggers," and cleavage at their target sites determines the phased register of the resulting phasiRNAs (Fei et al., 2013). A bias toward 22 nucleotide (nt) long RNAs has been observed for miRNAs that act as phasiRNA triggers; however, a two-hit mechanism has been proposed for 21 nt long miRNAs to trigger phasiRNA production [reviewed in Fei et al. (2013)].

The coding capacity of phasiRNA loci (*PHAS* loci; regions of phasiRNA production) has been underestimated, as demonstrated by Rajeswaran et al. (2012). Their results indicated that *PHAS* loci can have multiple triggers and their processing by dicer-like proteins (DCLs) can result in the production of a combination of 21 and 22 nt long phasiRNAs. Together, these features lead to new or shifted phased registers whose inclusion can greatly expand the repertoire of phasiRNAs produced from a given *PHAS* locus, with a commensurate increase in potential new targets (Fei et al., 2013). To highlight the recognition of shifted phased registers, we differentiate canonical and non-canonical phasiRNAs. Canonical phasiRNAs are 21 nt and produced strictly in a 21 nt register from the primary cutting site, the one giving rise to the most abundant number of phasiRNAs. Non-canonical phasiRNAs are those produced from alternative phased registers as well as all 22 nt long products. PhasiRNAs in the 21–22 nt size range act predominantly through PTGS; their role in gene regulation is currently an active field of research, and they function in development, defense, abiotic stress, and other biological processes (Wang and Chekanova, 2016).

Despite their significant regulatory potential, the understanding of miRNA- and phasiRNA-based gene regulation is limited. Genomic features or sequence signatures are not available to predict or detect *PHAS* loci, and current detection methods rely on sRNA expression data and the search for phased patterns in the distribution of sequenced sRNAs mapped to the genome or transcriptome (Guo et al., 2015). Expression of phasiRNAs has been shown to be inducible and dependent on specific stimuli (Komiya, 2017); therefore, a proper characterization requires an evaluation of data from multiple tissues, under different conditions and at different developmental stages.

In *A. thaliana*, multiple miRNA/phasiRNA modules have been described (reviewed in Fei et al., 2013; Wang and Chekanova, 2016). They are associated with cellular processes such as metabolic stress (Hu et al., 2011). However, there has been only one attempt to decipher genome-wide sRNA-mediated regulation (MacLean et al., 2010). These authors showed the potential for large scale sRNA-mediated regulatory networks, though their results were derived mostly from *in silico* predictions wherein the biological relevance had not been experimentally assessed. An important tool for validating predicted sRNA-transcript target interactions has been the development of degradome analyses (German et al., 2009). Degradome libraries capture the RNA products generated by sRNA targeting and cleavage of transcripts. By facilitating the experimental validation of the interactions in a high throughput manner, evaluations of sRNA activity and regulation through degradome analyses can be performed at a genome-wide level, resulting in networks of biological relevance.

The work described in this report was prompted by initial experiments designed to determine the plant sRNA-mediated response to virus infection. It became apparent that to identify changes in the plant sRNA response to virus (or any other biotic or abiotic stress), a global meta-network of interactions between sRNAs and transcribed RNA targets was required that could serve as reference for mapping changes. Changes in crop plant sRNA responses are being studied to improve crop productivity and better understand responses to drought (Zheng et al., 2019), nutritional stress (dos Santos et al., 2019), cold (Ghildiyal and Zamore, 2009; Bustamante et al., 2018; Zhu et al., 2019), and metal toxicity (Wang et al., 2019). Plant sRNAs regulate innate immunity to pathogens (Deng et al., 2018) and cross-kingdom RNA trafficking is being exploited as a crop protection strategy (Cai et al., 2018); the mechanism underlying these latter processes is sRNA-mediated RNA interference. The availability of a well-characterized network of sRNAs and transcribed RNA targets in the model plant *A. thaliana* will better enable progress for more applied studies in other plant species. Additionally, an expanded set of sRNA–mRNA interactions identified in studies such as this can facilitate approaches to metabolic pathway discovery using co-regulated (instead of colocalized) set of genes (Schläpfer et al., 2017). This latter suggestion stems from systems biology approaches for whole genome studies that have proven useful for the *de novo* identification of pathways and/or gene clusters, closing the genotype to phenotype gap (Ogura and Busch, 2016).

The objective of this study was to identify a comprehensive sRNA-mediated regulatory network at the genome-wide level in *A. thaliana* using a data-driven, degradome-supported bioinformatics analysis pipeline. This meta-network provides a reference frame for assessing sRNA-mediated regulation during growth, pathogenesis, and under different environmental conditions, and ultimately will reveal the role of sRNAs in the global genomic circuitry for the regulation of gene expression.

## Materials and Methods

### Experimental Design

Data were obtained by two methods: 1) all publicly available (NCBI) sRNA and degradome libraries from *A. thaliana* were compiled to provide a diverse representation of sRNA expression and regulation under varied conditions; these were derived from multiple tissues, developmental stages, and biotic and abiotic stress conditions; and 2) paired sets of sRNA-Seq and degradome data from aliquots of individual RNA extracts were produced as part of this study for 14 independent plant samples. There were four plant-virus treatments (described below) with four biological replicates per treatment for both sRNA-Seq and degradome analyses; two sRNA-seq libraries were low quality and removed. All the sRNA and degradome data from (1) and (2) were combined to identify an sRNA-mediated regulatory meta-network (described below).

### Plant Growth Conditions, RNA Extraction, and Library Preparation

Two-week-old *A. thaliana* Col. plants grown at 22 C with a 10 h photoperiod were mechanically inoculated with *Cucumber mosaic virus* or rubbed without virus as mock controls. (These treatments are from a separate study and the effect of virus is not addressed in this report.) Leaf tissue was collected 10 days post-inoculation, ground in liquid nitrogen, and total RNA extracted using Trizol (Thermo-Fisher) as recommended by the manufacturer. Each resulting RNA preparation was divided into two aliquots to be used as input for sRNA-Seq and degradome libraries. sRNA libraries were prepared from 1 μg of total RNA using methods described previously (Vargas-Asencio et al., 2017). For the degradome libraries, ~40 μg of total RNA was used. Degradome libraries were constructed using the method described by Zhai et al. (2014), but with the following modifications: a) different adapters and primer sequences were used (**Additional file 1: Table S1**), b) the PCR clean-up step was performed using Axygen™ AxyPrep Mag™ PCR Clean-up (Fisher) instead of Agencourt AMPure XP beads (Beckman Coulter), and c) EcoP151 (NEB) was used for the restriction enzyme digestion step instead of MmeI. Sequencing was performed using an Illumina Hiseq 4000 at the Genomics Resources Core Facility, Weill Cornell, NY, to obtain single-end 51-nt reads for both sRNA (accessions: SRR6234880- SRR6234893) and degradome libraries (accessions: SRR6235006- SRR6235021).

### Bioinformatics Tool for Identification of sRNA-Mediated Networks

A custom bioinformatics pipeline was implemented to identify sRNA-mediated networks. A detailed description is provided in the following sections. The overall strategy was to gather all available sRNA and degradome data, and to combine it with existing genome annotations and sRNA databases to produce a data-driven, degradome-supported network of interactions between sRNAs and transcripts. There are two types of nodes in the proposed network: sRNAs and transcripts. sRNAs include miRNA and phasiRNAs, and transcripts include miRNA precursors, *PHAS* loci, and mRNA transcripts targeted by sRNAs. Annotations are available for miRNAs, miRNA precursors, and potential target transcripts, while for *PHAS* loci, their sRNA triggers, and the resulting phasiRNAs, there are no genome-wide annotation available. The identification of these components and their interactions was therefore part of the tasks included in the pipeline. Newly generated annotations were combined with available genome and known miRNA annotations to perform a genome-wide-level search for sRNA–target interactions. Once all components and their interactions were identified and experimentally validated, they were consolidated into a network for downstream analysis.

### Reference Files and Datasets

The TAIR10 version for *A. thaliana* provided the reference genome (Swarbreck et al., 2008). Genome annotations were obtained from Araport11 (Cheng et al., 2017). Known miRNA and precursor sequences were obtained from miRBase (Kozomara and Griffiths-Jones, 2014) release 22. Gene ontology terms were obtained from Ensembl Genomes release 37 (Kersey et al., 2017).

Fourteen sRNA and 16 degradome libraries were produced in this study. These data were complemented with all publicly available sRNA datasets representing different tissues, stress conditions (biotic and abiotic), and developmental stages in *A. thaliana* (**Additional file 2: Table S2**), as well as all available degradome datasets (**Additional file 3: Table S3**).

### Data Processing

Reads (51 nt) from sRNA-Seq libraries were filtered using the adaptive adapter trimming function in Trim Galore (Kruger) to account for variability in library construction methodologies. Size was constrained to 20–40 nt after adapter trimming, and non-adapter containing reads were removed. Datasets were collapsed to unique sequences using the Fastx toolkit (Hannon); sequences with fewer than 50 reads were removed. Libraries containing less than 100 unique sequences were considered non-informative and removed. SRA degradome libraries were filtered using the adaptive adapter trimming function in Trim Galore with the minimum size after adapter trimming set to 18 nt. The resulting libraries were evaluated manually, and additional trimming was performed if there was evidence of remaining adapter sequences. For the libraries produced in this study, the first 6 nt derived from the library preparation process were removed. The Fastx toolkit was used to convert reads to fasta format.

### miRNA-*PHAS* loci-phasiRNA Annotation and Trigger Identification

*PHAS* loci detection was performed for each dataset using PhaseTank (Guo et al., 2015). Locus extension was set to zero, and the top 15% of regions with the highest accumulation of mapped reads (described as relative small RNA production regions in Guo et al., 2015) were analyzed for phasiRNA production. Results for all datasets were combined to produce *PHAS* loci with maximum length from overlapped results. Potential *PHAS* loci detected in less than 3 of the 902 libraries were discarded. The resulting loci were then extended by 220 nt on each side to perform a search for sRNA triggers associated with phasiRNA production.

PhasiRNA production triggers were searched using the degradome data. Thirty-nine degradome libraries were independently analyzed using CleaveLand4 (Addo-Quaye et al., 2009). Sequences from both strands of the extended *PHAS* loci were evaluated using known miRNAs as queries. A weighted scoring system (deg_score) to compile the independent degradome analysis results was developed as follows: cleavage events with degradome category zero per CleaveLand4 were given a score of 5, cleavage events with degradome category one were given a score of 4, cleavage events with degradome category two were given a score of 0.5. The scores for each event were added across all 39 degradome libraries. The highest scoring event per *PHAS* locus was selected as the initial phasiRNA triggering site; a minimum score of 10 was set to assigned triggers. When triggers were found, the polarity of the loci was set to the strand complementary to the triggers.

To identify the phasiRNAs produced by each *PHAS* locus sRNA reads from each library were mapped to the extended *PHAS* loci independently. No mismatches were allowed, sRNAs of 21 and 22 nt were accepted, counts for reads mapping to multiple locations were divided between the number of locations, reads with more than 10 mapping locations were removed, and reads mapping outside the original region (before extension) were not considered. Mapped reads were assigned to bins from 1 to 21 (phases) according to their mapping positions from the 5' end. Positions of reverse reads were shifted (+2) due to 3' overhang, to match forward read bin positions. The mapping was performed on each strand of the *PHAS* loci independently. A scoring system was developed to rank bins by read abundance for each locus across all sRNA libraries. The three most abundant bins per locus per library were used. The most abundant bin was given a score of 5, the second most abundant was given a score of 2, and the third most abundant was given a score of 0.5. The resulting scores from all libraries were added for each bin to produce a ranking of sRNA bins for each *PHAS* locus.

PhasiRNAs derived from miRNA triggering events were found by matching the phase register set by the degradome-confirmed miRNA triggering events to bin assignments. PhasiRNAs from immediately adjacent bins (-1, +1) were also collected. For the *PHAS* loci where no trigger was found, sRNAs from the most abundant bin and immediately adjacent bins were collected.

Resulting phasiRNAs were pooled with all known miRNAs to produce a new set of queries to search for phasiRNA production triggers using the degradome-based ranking strategy described above. To identify secondary and/or tertiary triggers, sRNAs whose cleavage events matched the polarity of the primary trigger (highest ranked, with score >10) were kept. The potential secondary/tertiary triggers were evaluated by matching their slicing site coordinates to those corresponding to the three most abundant sRNA bins per *PHAS* locus. Because 22-nt sRNAs were included in the analysis, which can alter the 21 nt phasing, the bins immediately adjacent (-1,+1) were also considered. In the cases where a match was found, the sRNAs were considered additional phasiRNA triggers. The assignment of secondary/tertiary triggers was further evaluated by determining if the phasiRNAs contained in the matched bins were biologically active (described below). PhasiRNAs derived from secondary and/or tertiary sRNA triggering events were found by matching the phase register set by the degradome-derived sRNA triggering events to bin assignments. The resulting phasiRNAs were pooled with known miRNAs to produce a final set of queries to search for phasiRNA production triggers using the strategy described above in this paragraph.

Corresponding trigger, *PHAS* locus and phasiRNA sets were evaluated and confirmed manually to produce a miRNA-*PHAS* loci-phasiRNA annotation. A novel nomenclature is proposed for phasiRNAs in order to provide consistent and detailed information about their biogenesis. To assign a *PHAS* loci to a gene ID, the *PHAS* loci with polarity assigned based on confirmed sRNA triggers were compared to the araport11 genome annotation, and if the locus had significant overlap (>70%) and matching polarity to annotated features (genes, transposons), the locus was assigned to the feature. If more than one feature matched a locus. If no trigger was found but the *PHAS* locus overlapped with an annotation, a tentative assignation notated with lowercase was used; if the *PHAS* locus did not match any annotation, the forward genomic orientation was kept and the loci were named using their coordinates. For phasiRNAs, they were named using the *PHAS* locus from which they derived, followed by up to four descriptors: 1) the number of registers (21 nt) from the 5' end of the transcript; 2) in parenthesis, offset to main phased register, if any; 3) polarity, a “+” was used if the phasiRNA derived from the mRNA strand or “-” if derived from the complementary sense strand; and 4) size was indicated in the case of 22 nt long phasiRNAs by adding “_22” to the end.

### Evaluation of *PHAS* Loci Characterization

To determine if the selection of canonical and non-canonical phased registers within phasi loci was adequate, all sRNAs (>50 copies per library) of 21 and 22 nt produced in this study that mapped to the *A. thaliana* genome were mapped to the regions where *PHAS* loci were detected. The mapped reads were evaluated according to whether their position corresponded to sites described in the resulting annotation.

### Proportion of sRNAs Identified Using the miRNA-*PHAS* Loci-phasiRNA Annotation

To evaluate the improvement in identification of sRNAs from sRNA-Seq datasets, all sRNAs of presumed biological relevance (e.g., with >50 copies per library) were identified using the annotation produced in this study. The relative abundance of the distinct types of sRNAs under consideration was evaluated based on the abundance of unique and total reads.

### Target Transcript Search and Characterization

The miRNA-*PHAS* loci-phasiRNA annotation was used to identify and quantify miRNAs and phasiRNAs as described above; an arbitrary threshold of 50 combined raw count was established to select candidates for transcript targets. Degradome datasets were analyzed independently using CleaveLand4 (Addo-Quaye et al., 2009) to find target transcripts for selected sRNAs. A custom scoring system (target_deg_score) was developed to evaluate the confidence and repeatability of sRNA–target transcript interactions. The following weighted scores were assigned to the degradome categories described in CleaveLand4 (Addo-Quaye et al., 2009): category 0 hits were given a score of 5, category 1 were given a score of 4, and category 2 were given a score of 0.5. Categories 3 and 4 were not considered informative. The scores were summed across all libraries for each cleavage event detected. An empirical cumulative distribution analysis was performed for the target_deg_scores and an arbitrary threshold of 15 was established to select for the 1% most reliable (high quality, most repeatable) sRNA–target transcript interactions.

For the curated set of sRNA–transcript pairs, the location of the target site in the transcript was converted to genomic coordinates using the GET map/cdna/:id/:region API from Ensembl Genomes (Kersey et al., 2017). Genomic features overlapping the resulting genomic coordinates were extracted and tabulated according to their abundance.

### Network Analysis and Visualization

For the sRNA-mediated regulatory meta-network, a bipartite directed meta-network was constructed by obtaining sRNAs, transcripts, and their interactions from a combination of existing miRNA and transcript annotations, the newly developed miRNA-*PHAS* loci-phasiRNA annotation, and the degradome search results from all datasets. Cytoscape (Shannon et al., 2003) was used for visualization and structural analysis. A functional characterization was performed using Bingo (Maere et al., 2005) to determine the representation of GO Slim categories of the genes included in the network as compared to the genome as a whole.

## Results

### *PHAS* Loci Detection

**Figure 1:**
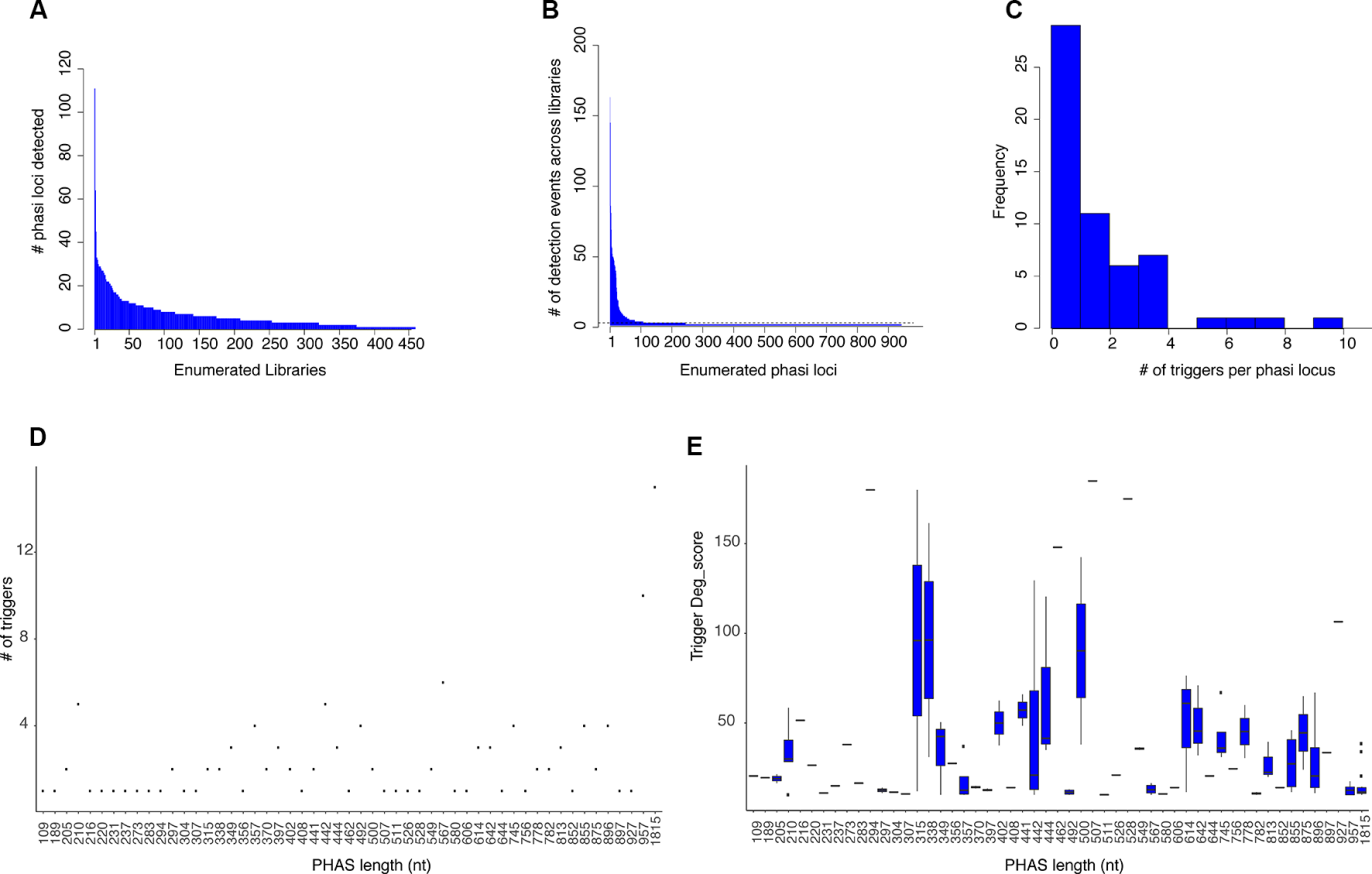
*PHAS* loci and phasiRNA triggers. **(A)** Histogram showing the number of *PHAS* loci detected per sRNA library across all libraries. Libraries are enumerated in the x axis; 902 libraries were evaluated and only those in which *PHAS* loci were found (n=426) are shown. The y axis shows the number of *PHAS* loci detected per library. **(B)** Histogram summarizing of recognition events (detection) of bona fide *PHAS* loci across all libraries. *PHAS* loci are enumerated in the x axis. The y axis shows the number of libraries in which a given *PHAS* locus was detected. Dotted line indicates the three detection events threshold utilized. **(C)** Distribution of number of phasiRNA production triggers in *PHAS* loci. **(D)** Degradome supported sRNA triggers. A Dot plot representation of the relationship between the number of degradome supported sRNA triggers and the length in nucleotides of the *PHAS* loci. **(E)** Boxplot representation of the degradome scores (deg_score) of identified sRNA triggers per *PHAS* loci length.

Currently there are no genomic features or sequence signatures that allow the identification of *PHAS* loci (regions of phasiRNA production) from a genome sequence; instead their detection depends on a search for phased patterns in sRNA-Seq data. Because of the observed variability in size between sRNA libraries and the assumption that phasiRNA expression may depend on specific environmental queues, the strategy used to identify *PHAS* loci was to independently evaluate sRNA-Seq datasets and then merge overlapping results to produce *PHAS* loci with maximum length. The number of consensus *PHAS* loci detected was variable, ranging from zero to more than 120 per library (**Figure 1A**).

A total of 942 *PHAS* loci were identified from the combined libraries (n=902; **Additional file 4: Table S4**). The consistency of *PHAS* loci detection was evaluated by determining the number of recognition events for each locus across all libraries. To remove spurious results, only *PHAS* loci detected in at least three libraries were included. The number of recognition events varied, with 107 *PHAS* loci independently detected in at least three libraries (**Figure 1B**). A failure to detect any given locus in a specific library could be due to expression limited to specific experiment conditions, e.g., stress, developmental stage, or tissue type or to a limitation in sensitivity.

### PhasiRNA Trigger Search

Once *PHAS* loci had been identified, a recursive method was designed to identify triggers by extending a search up and downstream of the detected *PHAS* loci, followed by searches for secondary or tertiary triggers that would explain the production of non-canonical phasiRNAs (22 nt long or derived from an alternative phased register). sRNAs were considered triggers if their predicted targeted position on the *PHAS* locus was consistent with the distribution of sRNAs and was supported by degradome data according to the quantitative criteria (deg_score) detailed in *Materials and Methods*. For the 107 *PHAS* loci evaluated, triggers were assigned for 57 of them. From the 108 unique sRNAs triggers identified, 16 corresponded to miRNAs and 92 were phasiRNAs; in some cases, sRNAs were assigned to multiple *PHAS* loci (**Additional file 5: Table S5**). Among the triggers, there were 16 canonical phasiRNA; 45 were 22 nt long, 64 were from a secondary phased register; 33 were both 22 nt long and derived from a secondary phased register. Consistent with Rajeswaran et al. (2012), multiple triggers per *PHAS* locus were detected in some cases (**Figure 1C**). The length of the *PHAS* locus was positively correlated with the number of putative triggers (R^2^ = 0.59, t = 5.54, df = 55, p < 0.01, **Figure 1D**); however, the magnitude of degradome support for putative triggers only showed a weak negative correlation with *PHAS* locus length (R^2^= -0.199, t = -2.8093, df = 190, p < 0.01, **Figure 1E**). Together these results indicate that even though more putative triggers were found for larger *PHAS* loci, the degradome support for sRNA triggers in larger *PHAS* loci was essentially retained.

### Genomic Features Assignment for *PHAS* Loci

To identify the genes or genome features corresponding to *PHAS* loci, regions containing *PHAS* loci were compared to the Araport11 (Cheng et al., 2017) genome annotation. An sRNA trigger acts on the transcribed strand of the *PHAS* locus, and when available, the polarity of the transcripts was inferred from the trigger assignments described in **Additional file 5: Table S5**. Knowledge of the transcript's polarity increased the confidence in the identification of the corresponding gene or feature and helped to resolve situations where annotated features existed for both genomic strands. Ninety-two of the 107 *PHAS* loci overlapped with annotated genomic locations, of which 49 were specifically assigned to genes based on their location and inferred strand polarity (**Table 1**). Six of the *PHAS* regions overlapped with two genomic features (when genes are present at the + and - strands) for a total of 98 genomic features that overlapped with the identified *PHAS* loci. These included: i) 41 *PHAS* loci overlapping with gene types previously shown to be involved in phasiRNA production, including six TAS genes, 20 (one pseudogene) Pentatricopeptide repeat/Tetratricopeptide repeat (PPR/TPR) protein genes, six F-box containing /Auxin response genes (ARFs), five (one pseudogene) nucleotide binding site-leucine rich repeat (NBS-LRR) domain containing genes (two *PHAS* loci were found in AT5G38850) (Wang and Chekanova, 2016), AGO1, a cation exchanger, a basic helix-loop-helix (bHLH) transcription factor and a Curculin-like (mannose-binding) lectin family protein (Chen et al., 2007; Xia et al., 2015); ii) six genes involved in gene expression regulation including DCL1, three GRAS family transcription factors, one LOB domain-containing transcription factor, and a DNA methyl transferase were found to produce phasiRNAs; iii) 24 genes involved in metabolism, structure, and other functions, with no previous connections to phasiRNAs; iv) 14 detected PHAS loci overlapping with 12 transposable elements (three *PHAS* loci were found in AT1TE51040); and v) a mixture of 13 overlapping sites, including five annotated as “long non-coding RNA,” four “Novel transcribed regions,” one “Other RNA,” and three with “natural antisense transcript”; these regions of phasiRNA production can be re-annotated as *PHAS* loci based on these results. There were 15 additional *PHAS* loci located in unannotated regions of the *A. thaliana* genome.

**Table 1:** Overlap of detected phasiRNA loci to genomic features (n=107).

	*PHAS* loci data				Overlapping genomic feature data				Overlap	Reference
	Chr	Start	End	Inferred polarity	Feature type	Feature polarity	GeneID	Feature annotation		
Trigger identified	Chr1	11454588	11454825	+	.	.	.		0	
	Chr2	5497	5801	+	.	.	.		0	
	Chr2	6461	8276	+	.	.	.		0	
	Chr3	14197115	14197304	+	.	.	.		0	
	Chr3	16158765	16159371	-	.	.	.		0	
	Chr4	1318879	1319142	-	.	.	.		0	
	Chr5	7006522	7007118	+	.	.	.		0	
	Chr5	11814198	11814509	+	.	.	.		0	
	Chr1	3945841	3946359	+	gene	+	AT1G11700	Senescence regulator	518	
	Chr1	4368802	4369096	-	gene	-	AT1G12820	Auxin signaling F-box 3	294	Howell et al. (2007)
	Chr1	4577301	4577793	-	gene	-	AT1G13360	Hypothetical protein	492	
	Chr1	7088193	7088490	-	gene	-	AT1G20450	Dehydrin family protein	297	
	Chr1	10472578	10473145	-	gene	-	AT1G29910	Chlorophyll A/B binding protein 3	567	
	Chr1	17203735	17203844	+	Transposable element	+	AT1G46120	Copia-like retrotransposon family	109	
	Chr1	17890967	17891581	-	gene	-	AT1G48410	ARGONAUTE 1 (AGO1)	614	Chen et al. (2007)
	Chr1	18549377	18549692	-	gene	-	AT1G50055	Trans-acting siRNA1b primary transcript (TAS1b)	315	Allen et al. (2005)
	Chr1	23177838	23178693	-	gene	-	AT1G62590	Pentatricopeptide repeat (PPR) superfamily protein	855	Howell et al. (2007)
	Chr1	23205212	23206109	-	gene	-	AT1G62670	Pentatricopeptide repeat (PPR) superfamily protein	897	
	Chr1	23299601	23300476	+	gene	+	AT1G62910	Pentatricopeptide repeat (PPR) superfamily protein	875	Howell et al. (2007)
	Chr1	23302103	23302999	+	gene	+	AT1G62914	Pentatricopeptide repeat (PPR) superfamily protein	896	
	Chr1	23307088	23307771	+	gene	+	AT1G62930	Tetratricopeptide repeat (TPR)-like superfamily protein	683	Howell et al. (2007)
	Chr1	23389496	23390241	-	gene	-	AT1G63080	Pentatricopeptide repeat (PPR) superfamily protein	745	Howell et al. (2007)
	Chr1	23413410	23414052	+	gene	+	AT1G63130	Tetratricopeptide repeat (TPR)-like superfamily protein	642	Chen et al. (2007)
	Chr1	23419941	23420383	+	gene	+	AT1G63150	Tetratricopeptide repeat (TPR)-like superfamily protein	442	Howell et al. (2007)
	Chr1	23451248	23451797	+	gene	+	AT1G63230	Tetratricopeptide repeat (TPR)-like superfamily protein	549	
	Chr1	23489425	23489630	-	gene	-	AT1G63320	Pentatricopeptide repeat (PPR) superfamily protein	153	
	Chr1	23490163	23490976	+	gene	+	AT1G63330	Pentatricopeptide repeat (PPR) superfamily protein	813	Howell et al. (2007)
	Chr1	23507868	23508766	+	gene	+	AT1G63400	Pentatricopeptide repeat (PPR) superfamily protein	898	Howell et al. (2007)
	Chr1	23987412	23987854	-	gene	-	AT1G64583	Tetratricopeptide repeat (TPR)-like superfamily protein	442	
	Chr2	8508	9465	+	gene	+	AT2G03875	Novel transcribed region	891	
	Chr2	11721669	11722113	-	gene	-	AT2G27400	Trans-acting siRNA1a primary transcript (TAS1a)	444	Allen et al. (2005)
	Chr2	15090887	15091257	+	gene	+	AT2G35945	Natural antisense transcript overlaps with AT2G35940	370	
	Chr2	16011479	16012261	+	gene	+	AT2G38230	Pyridoxine biosynthesis 1.1	782	
	Chr2	16537499	16538006	-	gene	-	AT2G39675	Trans-acting siRNA1c primary transcript (TAS1c)	507	Allen et al. (2005)
	Chr2	16539685	16540023	-	gene	-	AT2G39681	Trans-acting siRNA primary transcript (TAS2)	338	Allen et al. (2005)
	Chr2	18618970	18619432	-	gene	-	AT2G45160	GRAS family transcription factor	462	
	Chr3	5862034	5862383	+	gene	+	AT3G17185	Trans-acting siRNA primary transcript (TAS3)	349	Allen et al. (2005)
	Chr3	6915591	6915801	-	gene	-	AT3G19890	F-box family protein	210	
	Chr3	7795243	7796095	+	gene	+	AT3G22121	Natural antisense transcript overlaps with AT3G22120	852	
	Chr3	8529883	8530661	-	gene	-	AT3G23690	Basic helix-loop-helix (bHLH) DNA-binding superfamily protein	778	Chen et al. (2007); Xia et al. (2015)
	Chr3	9417547	9417820	-	gene	-	AT3G25795	Trans-acting siRNA primary transcript (TAS4)	270	Rajagopalan et al. (2006)
	Chr3	9870143	9870671	+	gene	+	AT3G26810	Auxin signaling F-box 2	528	
	Chr3	14200432	14202247	+	gene	+	AT3G06365	Novel transcribed region	1815	
	Chr3	22410991	22411491	-	gene	-	AT3G60630	GRAS family transcription factor	500	
	Chr3	23273360	23273801	-	gene	-	AT3G62980	F-box/RNI-like superfamily protein	441	
	Chr4	57957	58359	-	gene	-	AT4G00150	GRAS family transcription factor	402	
	Chr4	1472812	1473032	+	gene	+	AT4G04565	Long non-coding RNA	220	
	Chr4	1476283	1476590	+	gene	+	AT4G04595	Novel transcribed region	307	
	Chr4	5764837	5765363	+	gene	+	AT4G08990	DNA (cytosine-5-)-methyltransferase family protein	526	
	Chr4	8382142	8382898	-	Pseudogene	-	AT4G14610	pseudogene (CC-NBS-LRR class)	756	
	Chr4	10276479	10276990	-	gene	-	AT4G18670	Leucine-rich repeat (LRR) family protein	511	
	Chr4	17639712	17640120	-	gene	-	AT4G37540	LOB domain-containing protein 39	408	
	Chr4	18097248	18097605	-	gene	-	AT4G38770	Proline-rich protein 4	357	
	Chr5	5461590	5461946	+	gene	+	AT5G16640	Pentatricopeptide repeat (PPR) superfamily protein	356	
	Chr5	17566574	17567501	+	gene	+	AT5G43740	Disease resistance protein (CC-NBS-LRR class) family	927	
	Chr5	23394264	23394495	+	gene	+	AT5G57735	tasiR-ARF	231	Allen et al. (2005)
	Chr5	24309516	24309726	-	gene	-	AT5G60450	Auxin response factor 4	210	Allen et al. (2005)
No trigger identified										
	Chr1	24721142	24721509	.	.	.	.		0	
	Chr2	7349167	7349520	.	.	.	.		0	
	Chr2	7839895	7839958	.	.	.	.		0	
	Chr3	14199468	14199772	.	.	.	.		0	
	Chr3	17445687	17445934	.	.	.	.		0	
	Chr5	7683815	7684395	.	.	.	.		0	
	Chr5	22322745	22322921	.	.	.	.		0	
	Chr1	27833	28316	.	gene	+	AT1G01040	Dicer-like 1	483	
	Chr1	4185045	4185423	.	gene	-	AT1G12300	Tetratricopeptide repeat (TPR)-like superfamily protein	378	
	Chr1	4295826	4296206	.	gene	-	AT1G12620	Pentatricopeptide repeat (PPR) superfamily protein	380	
	Chr1	4354454	4355245	.	gene	+	AT1G12775	Pentatricopeptide repeat (PPR) superfamily protein	791	
	Chr1	6194911	6196018	.	gene	-	AT1G18000	Major facilitator superfamily protein	1107	
	Chr1	6200123	6201091	.	gene	+	AT1G18010	Major facilitator superfamily protein	968	
	Chr1	15464434	15465161	.	Transposable element	+	AT1TE51040	ATHILA6A	727	
	Chr1	15471434	15472100	.	Transposable element	+	AT1TE51040	ATHILA6A	666	
	Chr1	15485357	15486023	.	Transposable element	+	AT1TE51040	ATHILA6A	666	
	Chr1	21125812	21126104	.	Transposable element	-	AT1TE69815	VANDAL6	292	
	Chr1	23275517	23276374	.	Pseudogene	-	AT1G62860	pseudogene of pentatricopeptide (PPR) repeat-containing protein	857	
	Chr1	23386048	23386692	.	gene	-	AT1G63070	Pentatricopeptide repeat (PPR) superfamily protein	644	Howell et al. (2007)
	Chr1	23587585	23587805	.	gene	-	AT1G63615	Hypothetical protein	220	
	Chr1	23587585	23587805	.	gene	+	AT1G63630	Tetratricopeptide repeat (TPR)-like superfamily protein	220	
	Chr1	29427956	29428166	.	gene	-	AT1G09793	Long noncoding RNA	210	
	Chr1	29427956	29428166	.	gene	+	AT1G09797	Long noncoding RNA	210	
	Chr2	855647	856343	.	gene	-	AT2G02950	Phytochrome kinase substrate 1	696	
	Chr2	3251985	3252358	.	gene	-	AT2G07671	ATP synthase subunit C family protein	356	
	Chr2	3966746	3967025	.	Transposable element	-	AT2TE16865	ATHILA2	279	
	Chr2	11513043	11513358	.	gene	+	AT2G26975	Ctr copper transporter family	315	
	Chr2	13529851	13530171	.	gene	+	AT2G31820	Ankyrin repeat family protein	320	
	Chr3	343230	343814	.	gene	-	AT3G02020	Aspartate kinase 3	584	
	Chr3	3584608	3585005	.	gene	-	AT3G11410	Protein phosphatase 2CA	397	
	Chr3	4341697	4341988	.	gene	+	AT3G13370	Formin-like protein	291	
	Chr3	6524342	6524556	.	gene	-	AT3G18930	Transmembrane protein	214	
	Chr3	6524342	6524556	.	gene	+	AT3G18915	RING/U-box superfamily protein	214	
	Chr3	11983759	11983991	.	gene	+	AT3G00610	Novel transcribed region	232	
	Chr3	15677716	15678000	.	Transposable element	-	AT3TE63405	ATENSPM2	284	
	Chr3	17136708	17137721	.	gene	-	AT3G46550	Fasciclin-like arabinogalactan family protein	1013	
	Chr3	18733898	18734234	.	gene	+	AT3G50480	Homolog of RPW8 4	336	
	Chr4	3741606	3741942	.	Transposable element	-	AT4TE16565	ATHILA2	336	
	Chr4	4554898	4555171	.	Transposable element	+	AT4TE19135	ATENSPM3	273	
	Chr4	5567801	5567937	.	Transposable element	-	AT4TE23345	VANDAL21	136	
	Chr4	7890508	7890765	.	gene	-	AT4G13575	Hypothetical protein	257	
	Chr4	10180261	10180408	.	gene	-	AT4G06805	Long noncoding RNA	147	
	Chr4	10180261	10180408	.	gene	+	AT4G06810	Long noncoding RNA	147	
	Chr5	7684660	7684943	.	gene	-	AT5G22960	Alpha/beta-Hydrolases superfamily protein	283	
	Chr5	9789495	9789663	.	gene	-	AT5G27660	Trypsin family protein with PDZ domain-containing protein	168	
	Chr5	11850409	11850683	.	Transposable element	+	AT5TE42470	ATHILA6A	274	
	Chr5	12167704	12167920	.	Transposable element	+	AT5TE43315	ATHILA	216	
	Chr5	15555417	15556760	.	gene	+	AT5G38850	Disease resistance protein (TIR-NBS-LRR class)	1343	Howell et al. (2007)
	Chr5	15556984	15557720	.	gene	+	AT5G38850	Disease resistance protein (TIR-NBS-LRR class)	736	Howell et al. (2007)
	Chr5	15699210	15699441	.	Transposable element	+	AT5TE56690	RathE2_cons	231	
	Chr5	15757646	15758194	.	gene	+	AT5G39370	Curculin-like (mannose-binding) lectin family protein	393	Chen et al. (2007)
	Chr5	16640239	16640870	.	gene	-	AT5G41610	Cation/H+ exchanger 18	631	Howell et al. (2007)
	Chr5	16640239	16640870	.	gene	+	AT5G41612	Natural antisense transcript overlaps with AT5G41610	631	
	Chr5	17560854	17561363	.	gene	+	AT5G43725	Other RNA	509	
	Chr5	11778496	11778932	+	Transposable element	+	AT5TE42355	ATHILA2	436	
	Chr5	17560854	17561363	.	gene	+	AT5G43730	Disease resistance protein (CC-NBS-LRR class)	509	

### Interactions Between *PHAS* Regions

PhasiRNAs have the capacity to act as triggers to induce phasiRNA production in cis and/or trans by targeting their progenitor transcripts or unrelated transcripts, respectively, leading to regulatory modules that can involve multiple *PHAS* loci with the potential to integrate the regulation of phasiRNA production. Though a number of interactions of this kind (referred to herein as high-level interactions) have been described (Rajeswaran et al., 2012; Xia et al., 2017), a genome-level evaluation is not available.

**Figure 2:**
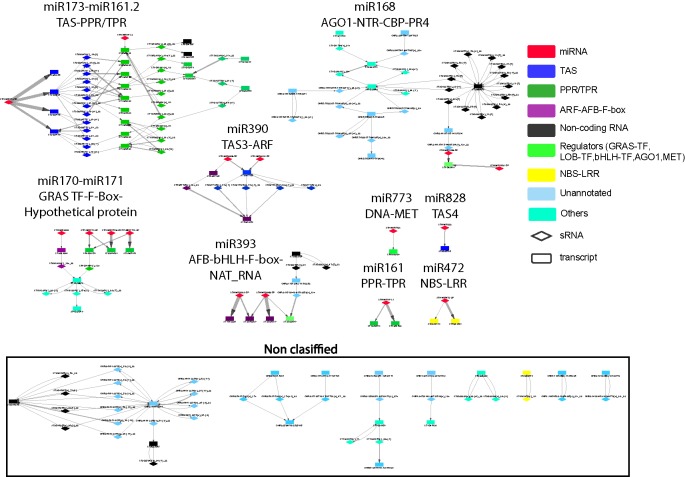
Network representation of interactions between phased interfering small RNA producing loci (*PHAS*) and small RNA (sRNA). *PHAS* loci connected by sRNA triggers were grouped into modules. Gene families previously reported as associated to *PHAS* loci and sRNA derived from these loci were colored as follows: miRNA (micro RNA) = red; TAS (Trans-acting small interfering RNAs) = dark blue, PPR/TPR (Pentatricopeptide/Tetratricopeptide repeat-like superfamily) = dark green; ARF-AFB-F-box (Auxin response factor/F-box containing protein) = purple, Non-coding RNA=black, Regulators=light green, NBS-LRR (Nucleotide binding leucine rich repeat protein) = yellow, genes/regions not previously associated to phasiRNA production or unannotated = light blue, Others=cyan. Diamonds represent sRNAs; rectangles represent *PHAS* loci. Non-coding RNAs include novel transcribed regions, natural antisense RNA, and long non-coding RNAs. Other acronyms are: GRAS TF=GRAS family transcription factor, AGO1=Argonaute 1 protein, MET= DNA (cytosine-5-)-methyltransferase family protein, LOB-TF= LOB domain-containing transcription factor, bHLH-TF= Basic helix-loop-helix (bHLH) DNA-binding superfamily protein transcription factor, CBP=chlorophyll A/B binding protein, NTR=novel transcribed region. The edge thickness between sRNAs and *PHAS* loci represents the degradome support for each interaction. Details for all of the node names in very small font can be in found in **Additional file 5: Table S5**, where they are listed individually within each of the 17 regulatory modules; alternatively they can be read within the (enlarged) online version.

To better understand these high-level interactions between *PHAS* loci, regulatory modules were constructed from *PHAS* loci connected by the phasiRNA triggering events tabulated in **Additional file 5: Table S5**. Seventeen regulatory modules of varying size and complexity were identified and are shown in **Figure 2**. Specific details on any component of the network can be found by cross-referencing **Figure 2** with the tabulated information in **Additional file 5: Table S5**. Consistent with the validity of the methodological approaches taken, the results included previously described interactions such as miR173-TAS1-TAS2-PPR/TPR (Allen et al., 2005; Howell et al., 2007), miR161-PPR (Howell et al., 2007), mir390-TAS3-ARF (Ozerova et al., 2013; Xia et al., 2017), miR393-AFB (Si-Ammour et al., 2011), miR828-TAS4 (Rajagopalan et al., 2006), miR472-NBS-LRR (Boccara et al., 2014), and miR168-AGO1 (Mallory and Vaucheret, 2009). The genome-wide approach used in this study allowed an extension of some of these modules. The miR173-TAS1/2-PPR/TPR was found to include seven additional PPR/TPR genes, a connection to miR161.2 and to interact with phasiRNAs-producing non-coding RNAs. A Basic helix-loop-helix (bHLH) type transcription factor and one non-coding RNA were linked to the miR393-AFB-F-box module, suggesting the existence of complex hormone-sRNA-TF regulatory interactions. The miR168-AGO1 (Mallory and Vaucheret, 2009) was extended to include interactions with non-coding RNAs, chlorophyll A/B binding proteins, and Proline-rich proteins 4. The miR2939-F-box module makes the third example of F-box containing regulatory modules, indicating an important connection between hormone and sRNA-mediated regulation. Two previously undescribed modules involving important transcriptional level regulators were identified. miR170-miR171-GRAS TF-F-box-LOB and miR773-DNA-MET provide further evidence for a direct connection between transcriptional and post-transcriptional regulation. Eight additional modules included combinations of non-coding RNAs, unannotated regions, and other genes not previously reported to produce phasiRNAs.

### Evaluation of *PHAS* Loci Characterization

**Figure 3:**
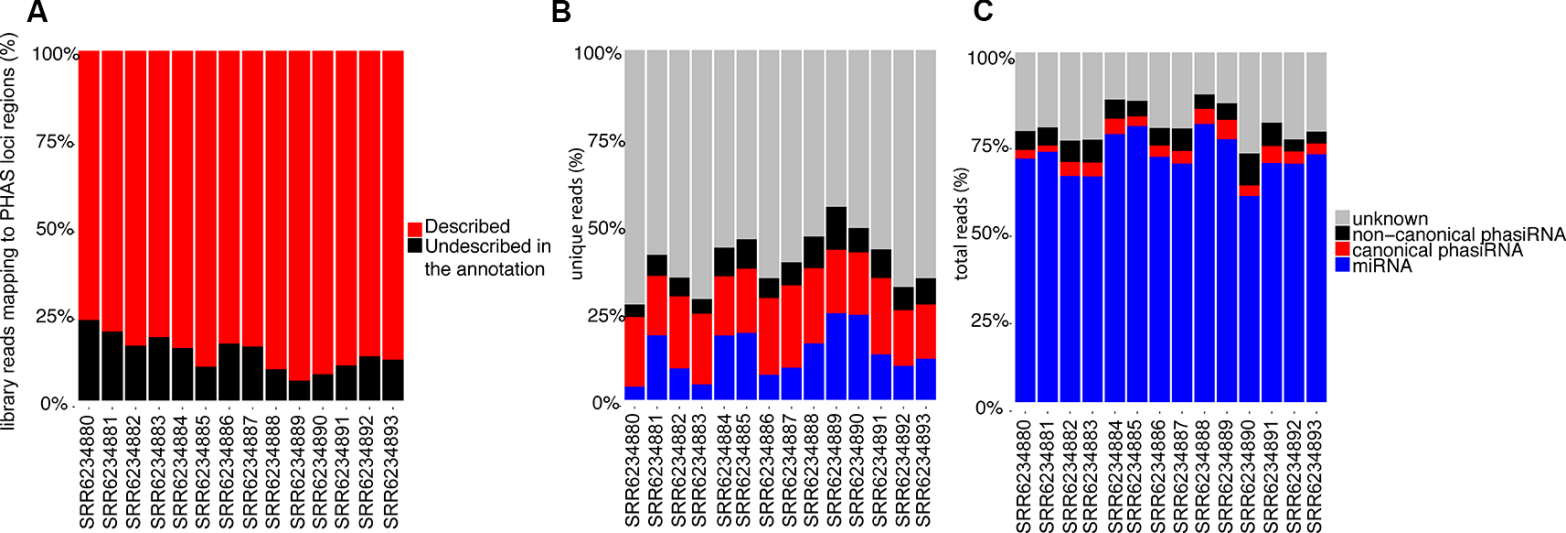
**(A)** Relative abundance of reads (>50 copies) mapping to *PHAS* loci that matched annotated phased registers. The category “Described in the annotation” indicates percentage of all reads mapping to regions where *PHAS* loci were detected that belong to the registers indicated in the annotation. **(B)** Relative abundance of unique 21 and 22 nt long sRNAs (>50 copies) based on their type, showing the relative proportion of sRNA types among unique reads. **(C)** Relative abundance of total 21 and 22 nt long sRNAs (>50 copies) based on their type, showing the sRNA types among all reads.

The identification of phased registers in *PHAS* loci is complicated by the possibility of multiple phasiRNA producing triggers and the presence of shift-inducing 22 nt long phasiRNAs. To evaluate the accuracy in phased register selection within *PHAS* loci, the relative abundance of reads mapping to the *PHAS* loci regions that match the selected phased registers was evaluated; assuming the abundance of sRNAs is indicative of biological activity, a high proportion of recalled sRNAs would indicate the correct registers were selected. Over 75% of all reads mapping to regions where *PHAS* loci were detected belong to the registers included in the annotation (**Figure 3A**). These results indicate that the annotation produced from a composite analysis of all available sRNAs libraries in *A. thaliana* resulted in an adequate representation of *PHAS* regions that can be used to successfully identify phasiRNAs in individual libraries.

### Proportion of sRNA Identified Using the miRNA-*PHAS* Loci-PhasiRNA Annotation

Multiple classes of sRNAs are known in *A. thaliana*, but an adequate annotation is only available for miRNAs (n=428); phasiRNAs have been reported sparsely, with Rajeswaran et al. (2012) providing the only case where phasiRNAs derived from the TAS1c locus are reported with details of their origin and phased register allowing their identification. Using the resulting miRNA-*PHAS* loci-phasiRNA annotation from our bioinformatics analysis, ~30 to 50% of all unique 21 and 22 nt long sRNAs from the sRNA-Seq datasets produced in this study were identified (**Figure 3B**); this nearly doubles in most cases the number of unique sequences identified, though a significant number remained unidentified. Evaluation of the total reads (**Figure 3C**) indicates that miRNAs make up the large majority of sRNA reads. There are ~10% of reads corresponding to phasiRNAs, both canonical and non-canonical, and ~15% of reads that remained unassigned.

### Experimental Support for sRNA Cleavage Activity

In plants, sRNAs act mainly through cleavage of their transcripts, yet there are examples of other mechanisms such as translational repression (Borges and Martienssen, 2015; Wang and Chekanova, 2016). Also, it has been shown that not all phasiRNAs produced from a *PHAS* locus are active; instead only some of them appear to be competent for loading into argonaute (AGO) containing complexes where they exert their activities (Fei et al., 2013). Therefore, in this study known miRNAs and phasiRNAs derived from the detected *PHAS* loci (including non-canonical phasiRNAs) were evaluated for biological activity using degradome data.

**Figure 4:**
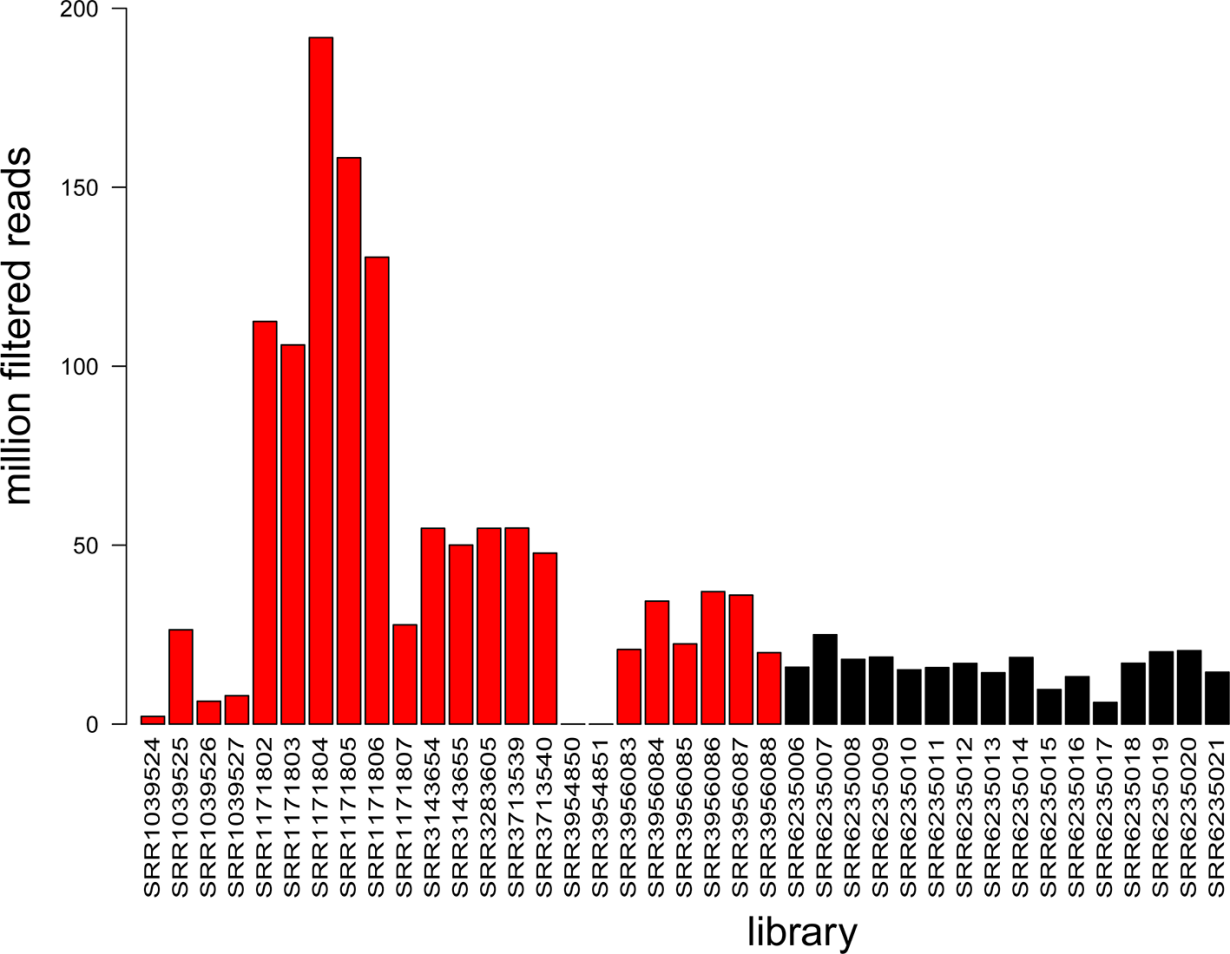
Summary of information available in degradome libraries. The histogram shows library yield as the number of million filtered reads for each of the 39 libraries. Colors: black refers to data produced in this study (16 libraries) and red refers to NCBI SRA data (23 libraries).

For *A. thaliana*, a limited number of degradome libraries are publicly available (**Additional file 3: Table S3**), including 11 datasets corresponding to inflorescence tissue, 6 to leaf tissue, 5 to seedling tissue, and 1 whole plant (Addo-Quaye et al., 2008; Creasey et al., 2014; Thatcher et al., 2015; Hou et al., 2016; Lin et al., 2017). Sixteen new degradome libraries from *Cucumber mosaic virus*-infected leaf tissue were produced as part of this study (accessions: SRR6235006-SRR6235021) and all available libraries were evaluated based on their yield (**Figure 4**). The data produced in this study represented a significant increase (~20%) in the total amount of degradome data available for *A. thaliana* in the NCBI SRA database.

**Figure 5:**
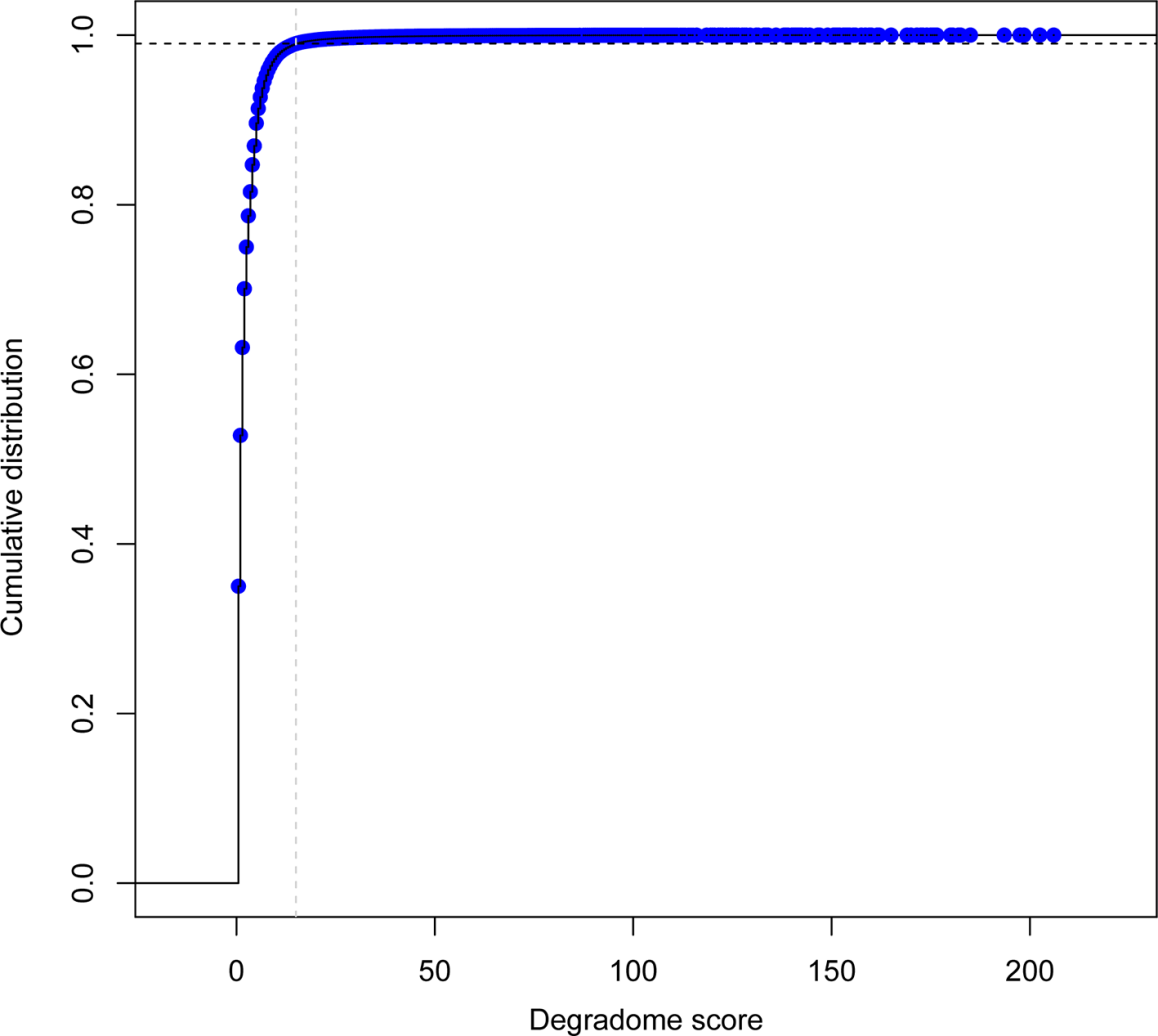
Empirical cumulative distribution function of degradome detection events per sRNA-transcript pair across all degradome libraries (n=39). Gray dashed line indicates the location for the degradome score (deg_score) value of 15; black dashed line indicates the corresponding 99% threshold.

To select for high confidence degradome supported sRNA–transcript interactions, quality and repeatability was evaluated using a custom scoring system (deg_score). The distribution of deg_scores for cleavage events was evaluated across all degradome libraries. Only the top 1% set of interactions (deg_score > 15) was considered for downstream analysis (**Figure 5**).

sRNAs were annotated as active if at least one of their predicted targets was confirmed (**Additional file 6: Table S6**). Experimental support was found for the targeting and cleavage activity of 201 out of 428 of annotated miRNAs, and the number of targets per active miRNA ranged from 1 to 46. In the case of phasiRNAs, 5047 out of 28,464 (~18%) were found to be active, and the number of targets per active phasiRNA ranged from 1 to 39.

**Figure 6:**
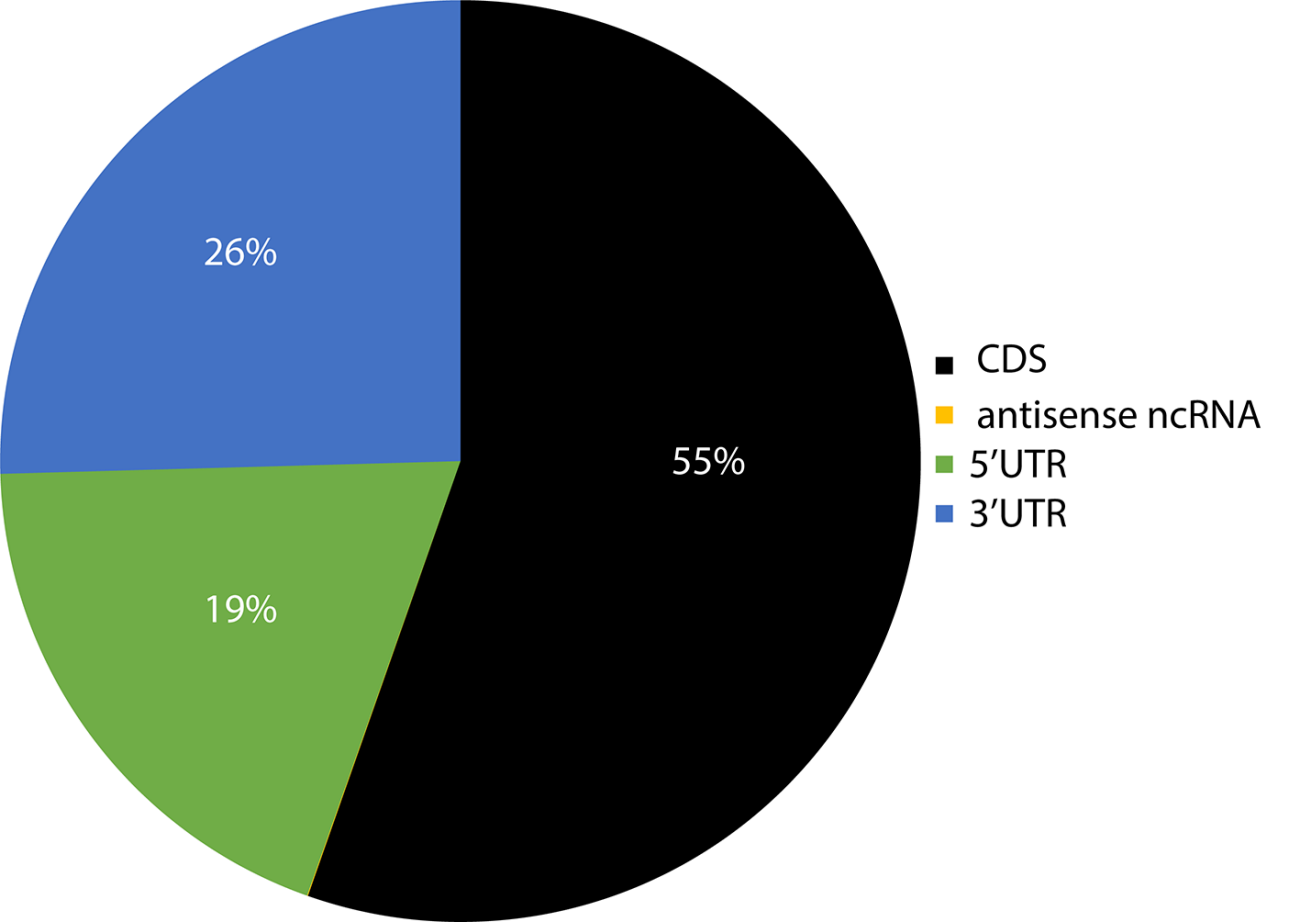
Pie chart representation of the relative abundance of sRNA target sites within different regions of their target's genes. The number in parenthesis indicates the total number of target counts for the respective region. CDS, coding sequence; UTR, untranslated region; ncRNA, non-coding RNA; lncRNA, long non-coding RNA; uORF, upstream open reading frame; snoRNA, small nucleolar RNA; snRNA, small nuclear RNA.

Previous studies on smaller datasets had reported the presence of sRNA targets in the 5'UTR (untranslated region), CDS (coding sequence), and 3'UTR regions of the genes (Kamthan et al., 2015). Given the significantly larger number of sRNA target sites identified here, the distribution of sites per region in the genes of target transcripts was evaluated to determine if there is a bias towards specific regions. CDS regions had the highest number of sRNA target sites, followed by 3' UTR and 5' UTRs (**Figure 6**). It should be noted that these results were not weighted to match the length of these gene features, and the observed distribution could be proportional to the total size of these features in the genome. A very small fraction (~0.03%) of target sites resided in non-coding RNAs.

With the additional degradome data from this study (**Figure 4**), this is the most comprehensive evaluation of sRNA cleavage activity to date for *A. thaliana*.

### Active PhasiRNA Characterization

**Figure 7:**
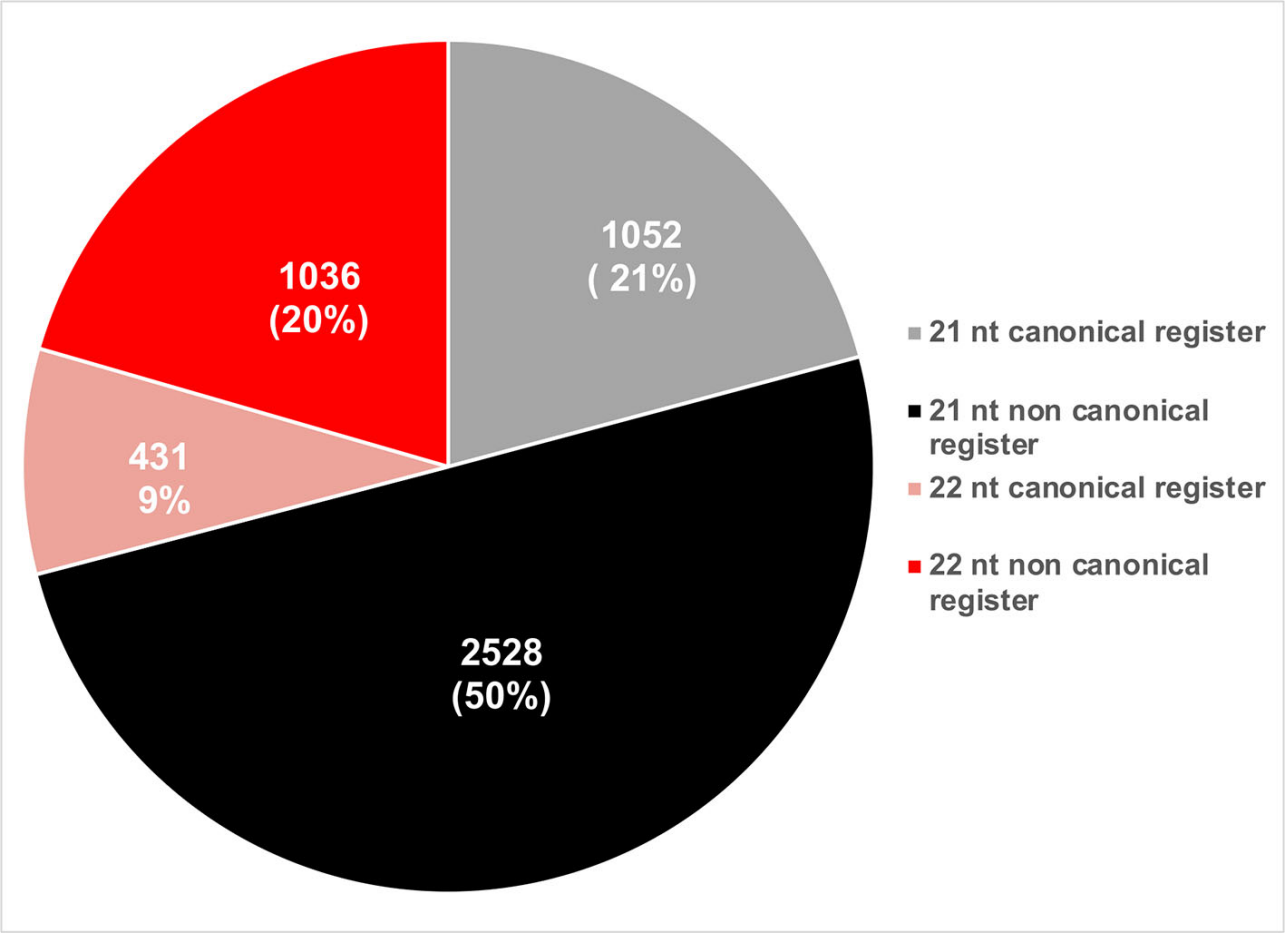
Pie chart representation of the relative abundance of phasiRNAs based on their size and phased register.

phasiRNAs whose activities were experimentally validated were evaluated based on their sizes and phased registers. Based on their size, a majority of active phasiRNAs were 21 nt long (~71%), with the remainder being 22 nt long phasiRNAs (**Figure 7**). phasiRNAs derived from non-canonical registers represent a significant majority in both size categories indicating the relevance of these sRNAs (**Figure 7**).

### Network Identification

**Figure 8:**
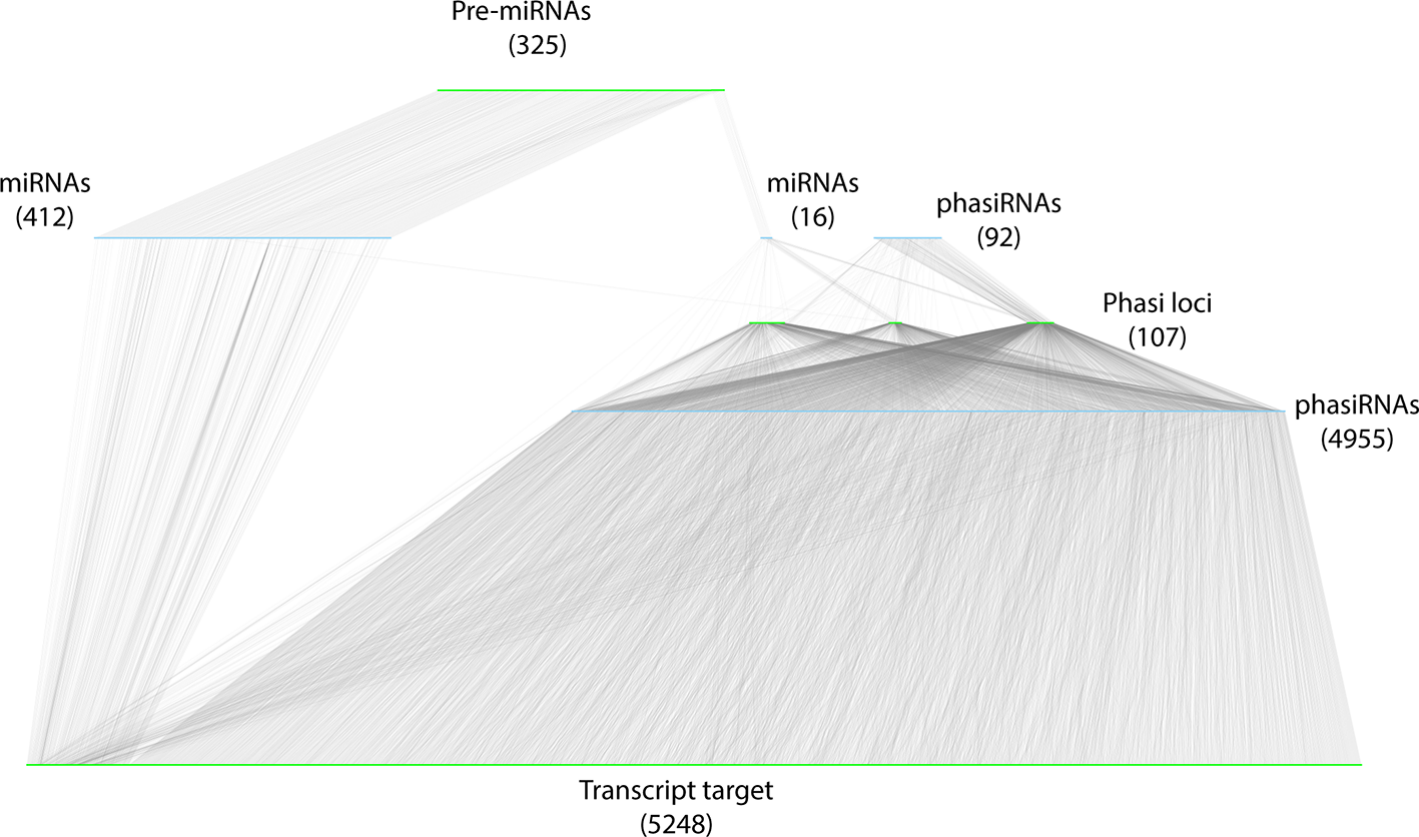
Representation of the resulting sRNA-mediated regulatory network. sRNA nodes are colored in light blue, transcripts are colored green, and edges are gray. The type and abundance are mentioned for each class of nodes. The network has been manually organized to reflect the biogenesis of sRNAs. Two sets of miRNAs (and numbers) are diagrammed: the miRNAs (412) that do not induce phasiRNA production, and those that induce phasiRNA production (15).

To integrate datasets and describe the *A. thaliana* sRNA-mediated regulatory network, a bipartite, directed network was constructed. To differentiate between the theoretical and functional (biologically relevant) components of the network, the network was restricted to the components identified in the miRNA-PHAS loci-phasiRNA annotation and interactions validated by degradome data. sRNA nodes (miRNAs and phasiRNAs) were restricted to those with known precursors and validated targets identified in degradome analysis. Transcript nodes included only those transcripts annotated as precursors (pre-miRNAs, *PHAS* loci) or validated in the degradome analysis to be direct targets of active sRNAs. The resulting network contained a total of 11,156 nodes, composed of 5,475 sRNA nodes (428 miRNAs and 5047 phasiRNAs) and 5,680 transcript nodes (325 miRNA precursors, 107 *PHAS* loci, and 5248 target transcripts). These nodes were connected by 13,160 edges; 5,602 of these were involved in the biogenesis of sRNAs and 7,558 edges were involved in sRNA cleavage of transcripts (**Figure 8**, **Additional file 7: Table S7**, **Additional file 8: Table S8**).

### Determination of the Regulatory Contribution of the sRNA-Mediated Network

**Figure 9:**
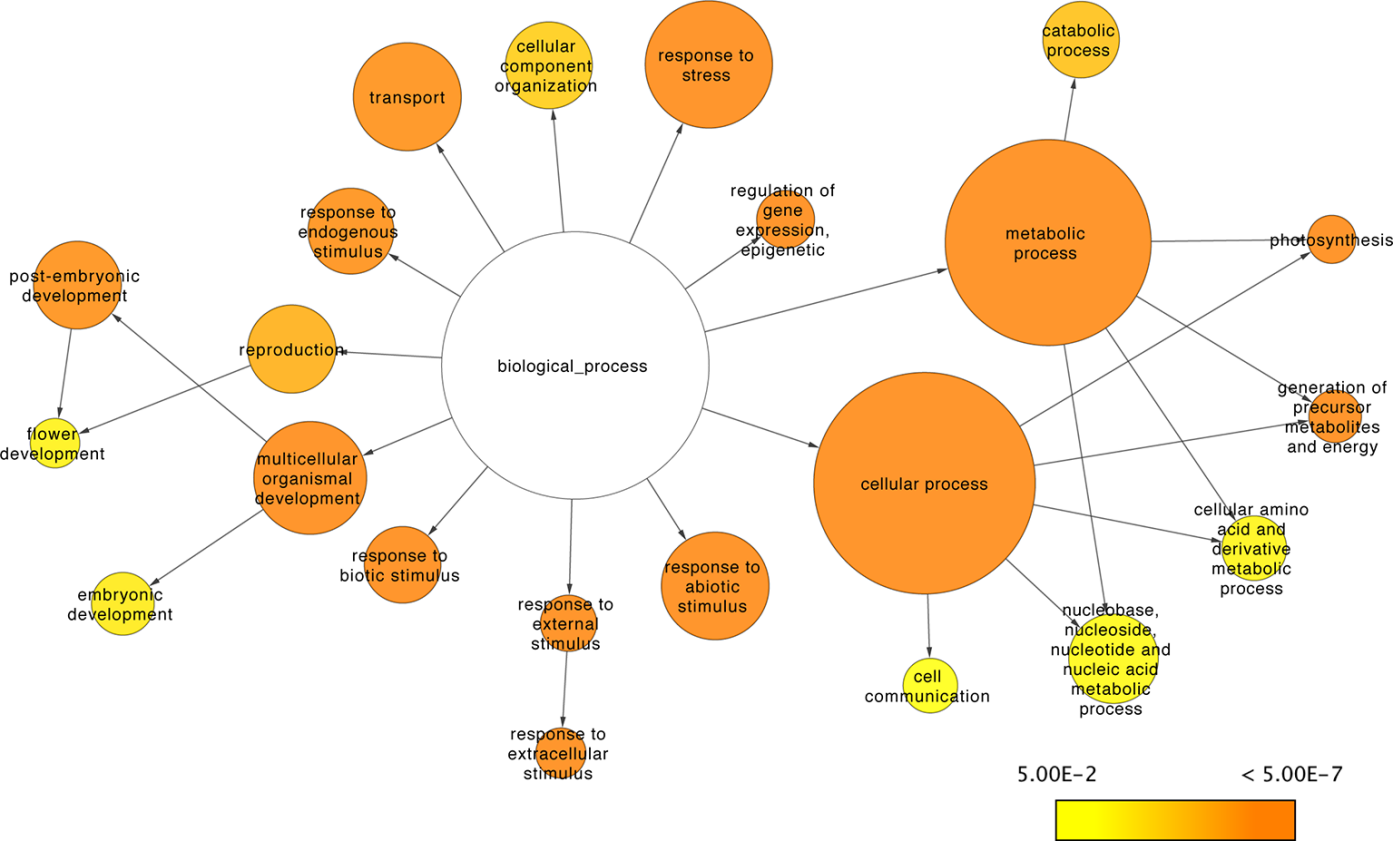
sRNA-mediated network functional analysis. Network representation of GO Slim categories showing enrichment for genes under sRNA regulation. Size of the nodes is proportional to the total amount of genes in the category; color scale indicates the corrected p-values for the enrichment test as described in Maere et al. (2005). Non-colored nodes are not significantly enriched (corrected p-value > 0.05).

Three metrics were used to assess the regulatory contribution of the resulting sRNA-mediated network. The proportion, function, and regulatory roles of the genes included in the network were evaluated. There are 33,341 *A. thaliana* genes defined in the araport11 genome annotation (Cheng et al., 2017). The proportion of annotated genes with degradome data to support interactions with sRNAs was about ~17% (n=5,624) of the total annotated genes. The networks regulatory role was assessed at a functional level using the GO annotations of the genes under sRNA control (i.e., with degradome supported interactions with sRNAs) to determine the biological processes in which sRNAs exert their control. Go slim annotations give a broad overview of the ontology content; using these terms, 22 of 45 functional categories under the biological processes domain were found to be disproportionally enriched in the network (versus a random distribution of categories among annotated genes) (**Figure 9**). Translation and RNA binding were the only terms out of 45 under the biological processes domain that were found to be underrepresented in the sRNA-mediated regulatory network (corrected p-value= 2.6977E-9 and 5.6680E-12, respectively).

Lastly, to assess the regulatory roles of the genes included in the network, the proportion of genes annotated to be involved in gene expression regulation (GO term: GO: GO:0003700) were evaluated for regulatory interactions with sRNAs. Based on GO annotation from Ensembl genomes release 37 (Kersey et al., 2017), 14% of genes (n=1039) involved in gene expression regulation are under sRNA control, including 21% (n=371) of annotated transcription factors (GO term: GO:0003700). Based on the proportion of total genes (~17%) interacting with sRNAs, the enrichment in the network of most categories under the biological processes domain (22/45), and the proportion of regulatory genes under sRNA control (14%), our results are consistent with the notion that sRNAs play a key regulatory role in plants.

### Structural Analysis of the sRNA-Mediated Regulatory Network

**Figure 10:**
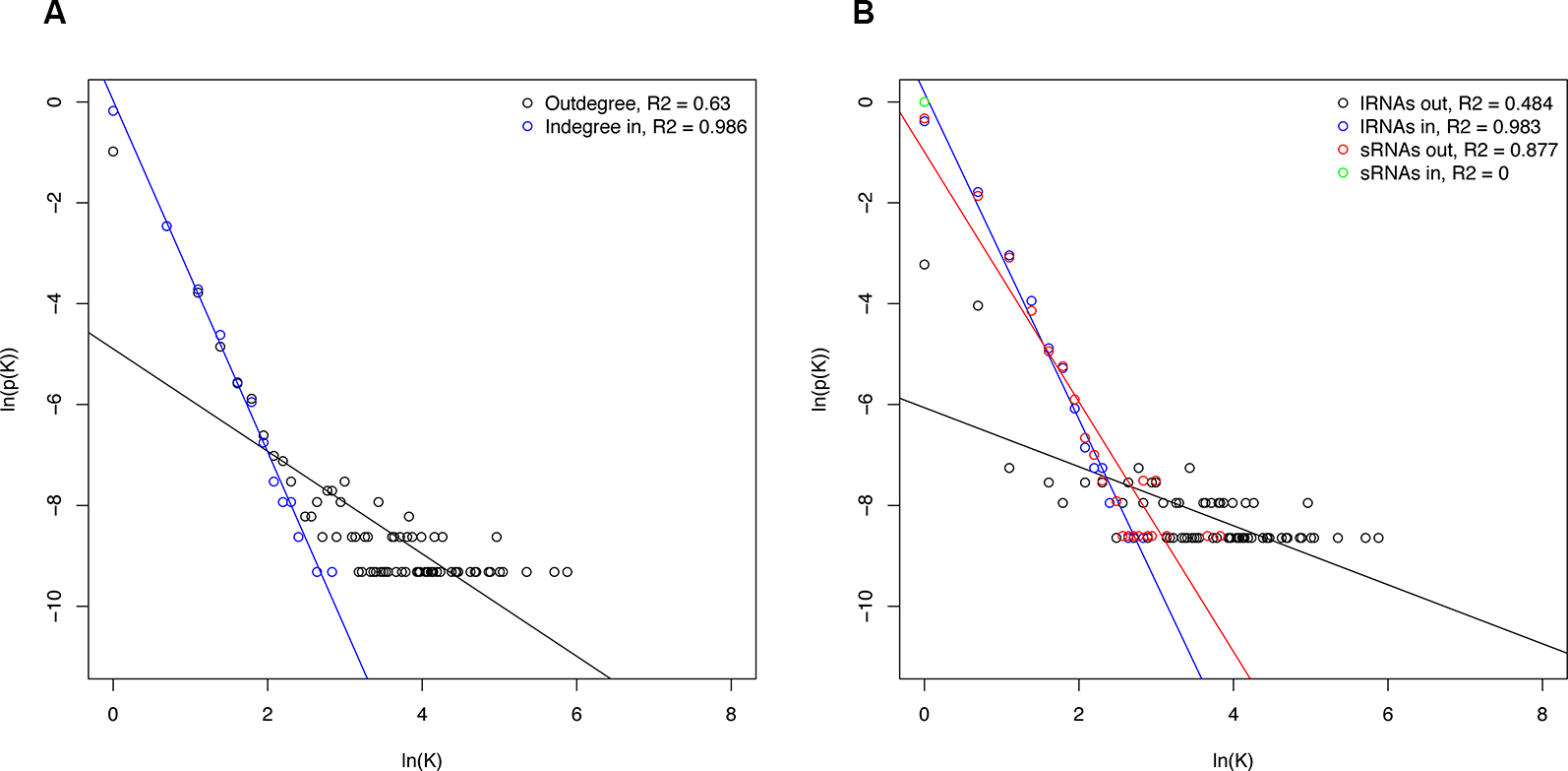
Degree distributions of sRNA-mediated network components. **(A)** Total degree distribution. **(B)** Degree distribution of the individual sRNA-mediated network components. Degree is represented by K and p(K) and is the number of nodes with degree K divided by total nodes. Regression lines for statistically significant correlations are shown. lRNA, long RNA (=transcripts).

Networks structure analysis allows a multiscale study of complex biological systems such as for the sRNA-mediated regulatory network presented here; global and local features can be identified and compared to related systems. To better understand properties of the resulting sRNA-mediated regulatory network and to determine the interconnectivity between miRNA and phasiRNA, structural features were analyzed. The sRNA-mediated regulatory network consisted of 192 disconnected components, or groups of connected nodes. Given the directed nature of the networks, each one of these corresponded to weakly connected components. The distribution of the number of nodes per component was uneven, with the largest component containing ~96% of the nodes, and the remainder consisting of 15 or less nodes. Network density, a measure of the ratio of the observed number of edges to the maximum number of edges, in this case was very low (<0.001), consistent with results from studies on other biological networks (Leclerc, 2008). By contrast, the clustering coefficient, a measure showing the tendency of a graph to be divided into clusters, was very low (<0.001), whereas in biological systems, higher values are usually observed (Pavlopoulos et al., 2011). In totality, the node degree showed a heavy-tailed distribution, both for indegree and outdegree (**Figure 10A**), describing a limited number of nodes with high degree while the majority have low degree. However, the bipartite nature of the network warrants a separate evaluation of the different types of interactions. Degree distributions for sRNAs and transcripts (lRNAs) were evaluated separately (**Figure 10B**). Negative correlations were found between node degree and abundance, except for the case of sRNA indegree as this indegree distribution is restricted by the nature of sRNA biogenesis and does not allow for testing.

## Discussion

The objective of this work was to identify a comprehensive, experimentally supported sRNA-mediated regulatory network at a genome-wide level. This required identifying the network components (pre-miRNA, miRNAs, *PHAS* loci, phasiRNAs and target transcripts) and the interactions between them, i.e., the nodes and edges of the network. MacLean et al. (2010) provided the first description of a broad level sRNA regulatory network in plants. Following this model and significant advancements in an understanding of sRNA biogenesis and activity (Rajeswaran et al., 2012; Fei et al., 2013; Wang and Chekanova, 2016), it became possible to investigate sRNA networks from a genomic view point, using only biologically relevant (experimentally supported) interactions. The miRBase database (Kozomara and Griffiths-Jones, 2014) and the araport11 genome annotation (Cheng et al., 2017) represent rich resources for mining miRNA precursors and mature sequences, gene transcripts, and *PHAS* loci. Using the existing model of sRNA biogenesis (Fei et al., 2013) and published bioinformatics tools (Guo et al., 2015), in combination with additional biogenesis features described by Rajeswaran et al. (2012), we designed an experimental approach and a bioinformatics analysis tool to perform a genome-wide identification of *PHAS* loci, their triggers, and resulting phasiRNAs. To account for the fact that sRNA production tends to be inducible and the expression can vary under different circumstances, a combination of libraries was employed. These included all sRNA libraries from the NCBI SRA database representing *A. thaliana* from multiple developmental stages, different tissues, and plants grown under varied biotic and abiotic stress conditions. The libraries produced in this study included plants with and without a biological stress (i.e., virus infection), conditions that will give rise to a varied sRNA response. Finally, to obtain a better view of sRNA cleavage activity on targeted transcripts, the degradome data available at NCBI for wild type *A. thaliana* was substantially expanded (~20%) with the libraries produced in this study. This allowed for the most comprehensive evaluation of the sRNA cleavage activity in *A. thaliana* to date, followed by the development of a genome-wide, experimentally supported sRNA-mediated regulatory network.

In order to accurately identify network components and their interactions, a number of factors were critical: a broader detection of *PHAS* loci at a genome-wide level, identification of non-canonical phasiRNAs, a newly designed strategy to assign *PHAS* triggers, and a significantly larger degradome dataset. The results from *PHAS* loci detection across combined sets of sRNA libraries made evident the need for the combinatorial approach used here in order to address the high levels of variability (**Figure 1A**). It was clear that individual libraries would fail to provide a representative view of *PHAS* loci, and that some regions only produced phasiRNAs under specific circumstances (**Figure 1B**). One hundred and seven *PHAS* loci were detected in at least three libraries, increasing the confidence of their assignment and resulting in a better definition of the 5' and 3' ends by combining overlapping loci into a maximum-length locus. Detection of previously described *PHAS* loci (Fei et al., 2013) was an indication of the accuracy of this strategy. Beyond the expected types of phasiRNA producing genes (TAS, PPRs, ARFs, and disease resistance), 69 new phasiRNA producing genes and non-annotated regions of the genome were detected as *PHAS* loci (**Table 1**), and these findings can be used to update and refine annotations for these regions. Three regions matched locations of natural antisense transcripts (AT2G35945, AT3G22121, AT5G41612), which have been reported to produce natural antisense small interfering RNAs (NAT-siRNAs) in a phased fashion (Borges and Martienssen, 2015).

As proposed by Rajeswaran et al. (2012) and using the biogenesis features described in their work, the inclusion of non-canonical phasiRNAs resulted in an expanded more comprehensive detection of miRNA–phasiRNA biogenesis cascades. Nearly 80% of the validated phasiRNAs were derived from an alternative phased register or were 22 nt long. Not all non-canonical phasiRNAs are novel; the most prominent case is a *TAS1c* derived phasiRNA, AT2G39675(-)_20-(+1) (described as “athTAS1c-D6(-)”), which has been shown to target its progenitor transcripts and trigger the productions of secondary phasiRNAs (Rajeswaran et al., 2012); it also acts in trans on other TAS transcripts. Despite the relevance of AT2G39675(-)_20-(+1) within the TAS-derived phasiRNA production cascades, it is not often appreciated that its location is shifted 1 nt with respect to the main 21 nt phased register set by the miR173 cleavage site. Moreover, the location is shifted by the production of a 22 nt phasiRNA in the previous register (Rajeswaran et al., 2012). Together, consideration of non-canonical phasiRNAs in this study provided a more accurate and comprehensive view of sRNA activity and regulatory potential.

Although there are limited reference points to determine the accuracy of assignment of secondary and tertiary triggers (Howell et al., 2007; Rajeswaran et al., 2012), the assignments of primary triggers in this study were consistent with previous reports (Howell et al., 2007; Fei et al., 2013). The composite use of degradome data allowed the identification of potential phasiRNA production triggers that act via cleavage of *PHAS* transcripts. One means of validating the bioinformatic approach taken in this study is to examine the extent to which these methods recapitulate recognized sRNA–*PHAS* loci interactions for which other published experimental evidence is available. The resulting network included the following previously reported examples: i) TAS1c-derived AT2G39675(-)_20-(+1) [described as “TAS1c 3'D6(-)”] triggering of phasiRNAs in TAS1a (AT2G27400) (Rajeswaran et al., 2012); ii) TAS2-derived AT2G27400(-)_19-(+1) [described as “TAS1a D9(-)”] triggering of phasiRNAs in PPR gene AT1G62590 (Howell et al., 2007); iii) TAS2-derived AT2G39681(-)_16-(+1), AT2G39681(-)_19-(2), and AT2G39681(-)_21-(2) [described as TAS2 3'D6(-), TAS2 3'D9(-), and TAS2 3'D11(-), respectively] triggering of phasiRNAs in 12 PPR/TPR genes (Table 1; Howell et al., 2007); and iv) TAS3-derived AT3G17185(+)_17+(-1) and AT3G17185(+)_18+(-1)_22 [described as atTAS3a-5'D8(+) and atTAS3a-5'D7(+), respectively] triggering of phasiRNAs in ARF4 (AT5G60450) (Allen et al., 2005). In most of these cases, both the 21 and 22 nt forms were identified as potential triggers (Table 2). Failure to detect previously reported interactions such as AT2G39675(-)_20-(+1) triggering in TAS1b (AT1G50055) and TAS1c (AT2G39675) (Rajeswaran et al., 2012) could be due to a non-cleavage based triggering or insufficient degradome evidence.

Beyond these previously reported examples, additional interactions were identified (**Additional file 5: Table S5**), including a TAS1a-derived AT2G27400(-)_20-(+1) triggering of phasiRNAs in TAS2 (AT2G39681), which represents a novel mechanism for the regulation of phasiRNA production in the TAS cascade. Of particular interest was the demonstration of the potential for connections between individual cascades wherein phasiRNAs from specific cascades can interact with other *PHAS* loci and trigger the production of downstream phasiRNAs, referred to herein as high-level interactions (**Figure 2**).

Due to the lack of reference in most cases, the validity of these triggers was additionally evaluated by testing the activity of their corresponding phasiRNAs. phasiRNAs produced by these secondary and tertiary triggers were shown to be functional. Degradome data was used to confirm their activity (discussed below), and additionally, non-canonical phasiRNAs were found to function as phasiRNA triggers, furthering the depth of the regulatory cascades. As described above, AT2G39675(-)_20-(+1), provides the best example of secondary triggers giving rise to phasiRNAs that then function as triggers anew. For the remaining 47% of *PHAS* loci with no assigned triggers, a number of them were found to overlap with regions annotated to produce NAT-siRNAs. The NAT-siRNA pathway has been shown to result in phased sRNAs, and this biogenesis mechanism (reviewed in Fei et al., 2013) is a likely explanation for some of the *PHAS* loci for which no sRNA trigger was found. Alternatively, the triggers were below the sensitivity of the methods used in this study and deeper sequencing and higher representation will be needed to find them.

To optimize the accuracy of the newly developed annotation of miRNA-phasiRNAs and produce a regulatory network of biological relevance, the biological activity of miRNAs and the expanded set of phasiRNAs was evaluated using degradome analysis. Using this enlarged dataset, targets were validated for ~47% of the predicted, annotated *A. thaliana* miRNAs. Approximately 17% of the detected phasiRNAs were found to be active. More than half of the degradome-validated phasiRNAs corresponded to non-canonical phasiRNAs further validating the accuracy of secondary and tertiary trigger assignments described above, and highlighting the relevance of these commonly overlooked phasiRNAs.

Restricting the network to interactions that were experimentally supported eliminated the problems associated with false positives in computational predictions, and provided a reference frame for functional, comparative, and structural analyses of biological relevance and applicability. The stringency and effectiveness of this approach is reflected by the significant reduction of the size of the network compared to MacLean et al. (2010); the number of sRNA nodes was reduced from ~40,000 to 5,475. Similarly, the number of transcripts [long RNAs (lRNA) in their study] was reduced from ~18,000 to 5,680. The number of edges was also reduced from ~38,000 “source” and ~140,000 target edges reported in MacLean et al. (2010) to 5,602 “source” (biogenesis related) edges, and 7,558 “target” (cleavage related) edges in this study, respectively. Contrary to network inference studies that mainly rely on co-expression patterns (see Albert, 2005 for review) and may be prone to false positives, a big data approach was taken in this study by aggregating all of the existing sRNA-Seq and degradome datasets to search for providing experimental evidence of sRNA and mRNA interactions. Given the uniqueness of this study and the limited number of positive controls, a conservative approach with very stringent thresholds was selected to filter interactions (see *Materials and Methods* for details), likely resulting in false negatives. We expect that this initial effort to define sRNA-based regulation will be revised and improved as more sequence information becomes available and complementary studies are conducted. Evaluation of this validated set of cleavage-related edges permitted an initial exploration of co-regulation between miRNA and phasiRNAs. miRNAs and phasiRNAs were found to be involved in co-regulation of ~8% of the target transcripts identified (**Figure 8**).

Cell functioning, behavior, and fate are controlled by the topology and dynamics of regulatory gene expression networks. Transcription factors and sRNAs appear to be the primary regulators in these networks (Cora et al., 2017), and technological and conceptual advances are making the study of gene regulation at this level possible. Yet in order to integrate these different regulatory systems, their individual roles and contributions must be established. In this study, limiting the sRNA-mediated network to validated interactions allowed for a realistic evaluation of its regulatory contribution. The evaluation of the network's regulatory contribution was performed at three different levels based on: i) the number of genes involved in the network, ii) the biological function of these genes, and iii) the interactions between sRNA and other gene expression regulators.

Close to 17% of *A. thaliana* genes were found to be under sRNA control in this study (**Figure 8**). Given the stringency of the analysis and assuming that neither the sRNA nor degradome dataset provided a complete representation of sRNAs and evidence of cleavage, it is reasonable to suggest that the network presented here should be considered a baseline representation of sRNA-mediated regulation that should be updated and revised as more data becomes available. Additionally, it should be noted that in the interest of considering only experimentally supported interactions, only cleavage-based regulation by sRNAs was considered here. Given that this appears to be the main mode of actions of sRNAs involved in PTGS in plants (Li et al., 2012; Borges and Martienssen, 2015; Wang and Chekanova, 2016), the presentation of regulatory interactions is likely to be representative. Research into alternative mechanisms of action can be expected to provide a better estimation of the regulatory contribution of sRNAs.

To further assess the regulatory contribution of the network, it was evaluated using the GO annotations of the genes included. At a GO slim level, ~50% of the biological processes were found to be disproportionally enriched in the network. These results are consistent with previous reports of sRNA regulatory roles in diverse processes such as response to stress (Liang et al., 2014), development (Nogueira et al., 2006), defense (Zhai et al., 2011), and other activities (Fei et al., 2013; Borges and Martienssen, 2015; Wang and Chekanova, 2016). While most biological processes were enriched in the network, we performed a search to determine if the opposite were true, whether some processes were underrepresented. Surprisingly, genes related to translation and RNA binding appear to have little to no regulation via sRNAs. While an explanation for this is not apparent, it suggests that there has been selection for the regulation of some biological processes by sRNA-independent mechanisms or at least with limited sRNA interactions.

sRNAs are known to be part of higher level regulatory circuits involving other factors involved in regulation at the transcriptional and posttranscriptional level (Walczak and Tkačik, 2011; Cora et al., 2017). These interactions were confirmed in this study, as ~14% of genes annotated to have a role in gene expression regulation were found to be under sRNA control. In particular, miRNA and transcription factors have been shown to act in coordination in other systems (Cui et al., 2007; Croft et al., 2012; Lin et al., 2012) to regulate common targets, as well as each other (), and transcription factors were well represented (21%) in the sRNA-mediated network. These results support the notion of crosstalk between regulatory factors and provide a reference frame to further investigate the regulatory circuits that control gene expression in plants. Quantitative estimates for the role of sRNAs in gene regulation in this study are consistent with the notion of sRNAs as “master regulators”.

The network representation of the sRNA-based expression regulation allows for a systematical characterization of its structural properties using complex network theory. Network topology analysis can be used to better understand the functional organization, underlying design principles, and organizing principles of biological networks (Steuer and López, 2007). Evaluation of network properties and topological features of empirically obtained biological networks revealed interesting commonalities between very distinct regulatory systems (Albert, 2005; Zhu et al., 2007; Pavlopoulos et al., 2011). As observed in other biological networks, the network was found to be sparsely connected, a feature that has been proposed to be evolutionarily selected to preserved robustness (Leclerc, 2008). Metabolic networks have been found to display, on average, higher clustering than random networks, reflecting their modular organization (Ravasz et al., 2002). Conversely, the sRNA-mediated network showed a low clustering coefficient. The bipartite nature of the network may obscure the detection of modules or more closely connected regions, and further characterization will be required to confirm observations on clustering. The degree distribution refers to the distribution of the number of edges connected to a node, and several examples of biological networks display heavy-tailed distributions (Albert, 2005). Overall the sRNA network node's degree showed heavy-tailed distributions, and negative correlations were found between node degree and abundance; this is a common situation in biological networks where highly connected nodes (main regulators or hubs) are rare, resulting in systems that display higher robustness towards random perturbations (see Albert, 2005 for review). These results are consistent with the previous study in *A. thaliana* using an *in silico* approach to model the interactions between sRNA and transcripts (MacLean et al., 2010). Given current models of sRNA biogenesis, their mode of action, and their function as part of a network, degree distributions should be further evaluated. The indegree distribution for sRNAs is restricted by their biogenesis; miRNAs and phasiRNAs are derived from specific precursors, which leads to an indegree fixed to one. The degree distribution of transcripts is also restrained: miRNA precursors often produced a single miRNA limiting their outdegree, while target transcripts are inherently terminal nodes with zero outdegree. Altogether, the resulting sRNA-mediated network showed similar structural features to other biological networks.

## Conclusions

Individual phasiRNA cascades have been studied in some detail (Allen et al., 2005; Rajagopalan et al., 2006; Rajeswaran et al., 2012), but a genome-wide view to determine if these cascades correspond to independent modules or if they act together within a larger regulatory network remains an open question. Detailed descriptions of the TAS cascade system provided an indication of the potential for interconnectivity. In this work, the construction of a genome-level network of interactions between sRNAs and transcripts that includes phasiRNA biogenesis and sRNA cleavage activity allowed a visualization of high-level interactions of phasiRNA regulatory cascades. Additionally, a coregulation of transcripts by multiple sRNAs from different sources became apparent. Using a network approach on sRNA biogenesis, a high level of interconnectivity was observed between cascades, resulting in large regulatory modules with potential cross regulation of phasiRNA production. At the transcript targeting level, it was found that a large proportion of sRNA–transcript interactions (>95%) were part of a major (weakly) connected network, indicating a considerable level of connectivity; this suggests that miRNAs and multiple phasiRNA cascades act in coordination in the co-regulation of gene expression.

## Data Availability Statement

Publicly available datasets were analyzed in this study. This data can be found here: https://www.ncbi.nlm.nih.gov/sra.

## Author Contributions

JV-A designed the project, performed all the experiments, performed the computational analysis, data analysis and interpretation, and wrote the manuscript. KP contributed to design of the project, data interpretation, manuscript writing, and critical revision. All authors read and approved the final manuscript.

## Funding

Funding for this research was provided by the College of Agriculture and Life Sciences, Cornell University.

## Conflict of Interest

The authors declare that the research was conducted in the absence of any commercial or financial relationships that could be construed as a potential conflict of interest.

## Abbreviations

DCLs, Dicer-like proteins; GO, gene ontology; miRNA, microRNA; phasiRNAs, phased small interfering RNAs; *PHAS*, phasiRNA loci; sRNA, small RNA.
